# Monitoring of Non-β-Lactam Antibiotic Resistance-Associated Genes in ESBL Producing *Enterobacterales* Isolates

**DOI:** 10.3390/antibiotics9120884

**Published:** 2020-12-09

**Authors:** Pottathil Shinu, Rajesh Bareja, Anroop B. Nair, Vashishth Mishra, Snawar Hussain, Katharigatta N. Venugopala, Nagaraja Sreeharsha, Mahesh Attimarad, Sahibzada Tasleem Rasool

**Affiliations:** 1 Department of Biomedical Sciences, College of Clinical Pharmacy, King Faisal University, Al-Ahsa 31982, Saudi Arabia; shhussain@kfu.edu.sa (S.H.); srasool@kfu.edu.sa (S.T.R.); 2 Department of Microbiology, Faculty of Medicine, Government Medical College and Hospital, Badaun 243601, India; rajeshbareja@gmail.com (R.B.); vashishtmmishra38@gmail.com (V.M.); 3 Department of Pharmaceutical Sciences, College of Clinical Pharmacy, King Faisal University, Al-Ahsa 31982, Saudi Arabia; anair@kfu.edu.sa (A.B.N.); kvenugopala@kfu.edu.sa (K.N.V.); sharsha@kfu.edu.sa (N.S.); mattimarad@kfu.edu.sa (M.A.); 4 Department of Biotechnology and Food Technology, Durban University of Technology, Durban 4001, South Africa; 5 Department of Pharmaceutics, Vidya Siri College of Pharmacy, Off Sarjapura Road, Bangaluru 560035, India

**Keywords:** *Enterobacterales*, extended spectrum β-Lactamase, plasmid mediated quinolone resistance, aminoglycoside modifying enzymes, trimethoprim/sulfamethoxazole resistance, Gram-negative bacteria

## Abstract

Genetic context of extended spectrum β-Lactamase (ESBL) producing *Enterobacterales* and its association with plasmid mediated quinolone resistance (PMQR), aminoglycoside modifying enzymes (AME) and Trimethoprim/Sulfamethoxazole (TMP-SMX) resistance is little known from North India. Therefore, the current study was aimed to investigate the frequency of Non-β-Lactam antibiotic resistance associated genes in extended spectrum β-Lactamase producing *Enterobacterales*. For this study, Non-Duplicate phenotypically confirmed ESBL producing *Enterobacterales* isolates (N = 186) were analyzed for ESBLs, PMQRs, AMEs and TMP-SMX resistance genes using polymerase chain reaction (PCR). PCR detected presence of PMQR genes in 81.29% (N = 139) of ESBL isolates (N = 171), AME genes in 60.82% and TMP-SMX resistance genes in 63.74% of the isolates. Molecular characterization of ESBL producing *Enterobacterales* showed 84.79% *bla_TEM_* followed by 73.68% *bla_CTX-M_*, 43.86% *bla_SHV_*, 19.88% *bla_PER_ and* 9.94% *bla_VEB_*, respectively. Analysis of PMQR genes revealed 77.7% *aac(6′)-lb-cr* the most commonly detected gene followed by 67.63% *oqxB*, 62.59% *oqxA,* 43.17% *qnrB*, 19.42% *qnrD,* 18.7% *qnrS*, 9.35% *qnrA*, 3.6% *qepA* and 2.88% *qnrC*, respectively. Analysis of AMEs gene profile demonstrated 81.73% *aac(6′)-Ib*, the most frequently encountered gene followed by 46.15% *aph(3′)-Ia,* 44.23% *ant(3”)-Ia,* respectively. A 100% prevalence of *sul1,* followed by *dfrA* (54.63%) and *sul2* (15.74%) was observed. In summary, prevalence of ESBL-Producing genes (particularly *bla_TEM_* and *bla_CTX-M_*) along with PMQR, AMEs, and TMP-SMX resistant genes may potentially aid in the transfer of antimicrobial resistance among these strains.

## 1. Introduction

β-Lactam antibiotics are used for treating most of the human infections that are caused by Gram-negative bacteria belonging to the family *Enterobacterales* [1]. However, the use of β-Lactam antibiotics is challenging due to the emergence of various antimicrobial resistance (AMR) mechanisms particularly the presence of extended-spectrum β-lactamases (ESBLs) [2]. ESBLs are considered the most extensively encountered AMR mechanisms prevalent in *Enterobacterales* and other clinically significant Gram-negative bacteria [1,2]. Genes coding for ESBLs production are often located on plasmids that also carry genes coding for resistance to fluoroquinolones, aminoglycosides, trimethoprim- sulfamethoxazole (TMP-SMX) [1]. Further, a high level of fluoroquinolone resistance was reported among ESBLs producing *Enterobacterales* [2,3]. Chromosomal mutations and plasmid-mediated quinolone resistance (PMQR) are considered the most relevant mechanisms of fluoroquinolone resistance among *Enterobacterales* [4,5]. Generally, PMQR determinants produce low-level of quinolone resistance, however, reports reveal that the presence of PMQR determinants may enhance the degree of chromosomal mediated quinolone resistance if present in the same strain [3,6,7,8]. PMQRs are mainly categorized into three groups; (i) quinolone resistance (*qnr*) gene mediated (ii) quinolones modifying aminoglycoside acetyltransferase encoding genes (*aac(6′)-Ib-c*), (iii) plasmid-mediated quinolone efflux pumps *qepA, oqxA*, and *oqxB* [6].

Literature indicates that synthesis of aminoglycoside modifying enzymes (AMEs) is an important AMR mechanism that produce high level of aminoglycoside resistance among Gram- negative bacteria [9,10]. These AMEs are grouped into three categories: (i) aminoglycoside N-acetyltransferases (AACs), (ii) aminoglycoside O-phosphotransferases (APHs), and (iii) aminoglycoside O-nucleotidyltransferases (ANTs) [10]. Further, the structural genes coding for AMEs are often located on plasmids that carry multiple resistance elements for ESBLs [11,12]. This type of association between ESBL and AMEs coding genes are of foremost apprehension in the treatment of bacterial infections. Besides resistance to β-lactams, fluoroquinolones, and aminoglycosides, members of *Enterobacterales* demonstrate resistance to TMP-SMX as well. However, little has been reported on the genetic context of various species of ESBL producing *Enterobacterales* and its association with, PMQR, AMEs TMP-SMX resistance exclusively from North India. Therefore, the current study was designed to analyze the distribution of non-β-lactam antibiotic resistance associated genes prevailing in ESBL producing *Enterobacterales.* To our knowledge, this is the first study that investigated the distribution of AME, PMQR and TMP-SMX resistance genes among ESBL producing *Enterobacterales* that are isolated from North India. Furthermore, this study included clinical isolates of different members of *Enterobacterales (Escherichia coli*, *Klebsiella pneumonia*, *Citrobacter freundii, Klebsiella oxytoca, Morganella morganii Proteus mirabilis, Proteus vulgaris,* and *Enterobacter cloacae),* while the earlier studies limited their molecular characterization in two species (*E. coli* and *K. pneumoniae)* [8,10,13]. In addition, this study explores prevalence of TMP-SMX resistance genes among ESBL producing clinical *Enterobacterales*, an observation that has been made only a few times earlier.

## 2. Results

Among the total phenotypically confirmed ESBL isolates (N = 186) obtained, *K. pneumoniae* 16.13% (30/186), *E. coli* 9.67% (18/186), *K. oxytoca* 2.15% (4/186), *P. mirabilis* 1.61% (3/186) and *P. vulgaris* 0.53% (1/186) were resistant to all the antibiotics tested. Table 1 shows distribution of antibiotic susceptibility pattern of various antibiotics tested against different strains of *Enterobacterales*. The highest antibiotic resistance rate was noted for ampicillin 95.89% (70/73) followed by TMP-SMX 90.86% (169/186). The resistance rate of β-lactams were: cefazolin 94.08% (175/186), amoxicillin-clavulanic acid 77% (124/161), cefotaxime 82.8% (154/186), cefepime 66.67% (124/186), ceftazidime 89.78% (164/186), ceftriaxone 88.17% (164/186), cefoxitin 76.83% (126/164), cefpodoxime 86.11% (155/180), cefuroxime 86.02% (160/186), and ceftizoxime 83.3% (155/186), respectively. The resistance rate of fluoroquinolones were 75.27% (140/186), 69.89% (130/186), 76.88% (143/186), 75.27% (140/186), and 71.51% (133/186) for ciprofloxacin, levofloxacin, nalidixic acid, gatifloxacin, and moxifloxacin, respectively. Similarly, the resistance rate of aminoglycosides were 64% (119/186), 61% (114/186), 49% (91/186), and 59% (109/186) for gentamycin, tobramycin, amikacin, and kanamycin, respectively. The lowest resistance rates were associated with imipenem 43.01% (80/186) and meropenem 46.77% (87/186), respectively (Table 2 and Supplementary Materials Table S1).

Table 3 demonstrates distribution of PMQR, AME, and TMP-SMX resistance genes in *Enterobacterales*. Among the total phenotypically confirmed ESBL isolates (N = 186), PCR confirmed presence of ESBL genes (*bla_CTX-M_, bla_TEM_, bla_SHV_, bla_PER_ and bla_VEB_*) in 91.94% (171/186) of *Enterobacterales* isolates. However, genes coding for ESBL production were not detected from *K. pneumoniae* 2.69% (5/186), *E. coli* 2.69% (5/186), *K. oxytoca* 1.07% (2/186), *P. mirabilis* 1.07% (2/186), *C. freundii* 0.54% (1/186) and therefore, these isolates were not included for further analysis. ESBL genes were detected from pus (N = 97), respiratory tract specimens (N = 44), blood and body fluids (N = 30), respectively (Supplementary Materials Table S2). The detected ESBL genes (91.94%) were distributed in different members of *Enterobacterales* as follows; *K. pneumoniae* 40.35% (69/171), *E. coli* 30.99% (53/171), *P. mirabilis* 7.6% (13/171), *C. freundii* 7.02% (12/171), *K. oxytoca* 5.2% (9/171), *E. cloacae* 5.2% (9/171), *P. vulgaris* 1.75% (3/171), and *M. morganii* 1.75% (3/171), respectively. Of the 91.94% (N = 171) genotypically confirmed isolates, *bla_TEM_* 84.79% (145/171) was the most common ESBL gene followed by *bla_CTX-M_* 73.68% (126/171), *bla_SHV_* 43.86% (75/171), *bla_PER_* 19.88% (34/171) and *bla_VEB_* 9.94% (17/171), respectively.

Among the total phenotypically confirmed ESBL isolates (N = 186), 81.72% (152/186), 70.43% (131/186), and 90.32% (168/186), were resistant to fluoroquinolones, aminoglycosides and TMP-SMX, respectively. Out of the 91.94% (171/186) of the genotypically confirmed ESBL strains, PCR could detect 81.29% (PMQR), 60.82% (AME), 63.74% (TMP-SMX) of genes in these strains. Of the total PMQR genes (81.29%) detected, the distribution of genes in different members of *Enterobacterales* (N = 139) as follows; *K. pneumoniae* 44.6% (62/139), *E. coli* 31.65% (44/139), *P. mirabilis* 8.63% (12/139), *C. freundii* 5.03% (7/139), *K. oxytoca* 5.03% (7/139), *E. cloacae* 2.16% (3/139), *P. vulgaris* 2.16% (3/139), and *M. morganii* 0.72% (1/139), respectively (Table 3). PMQR genes were detected from wound (84/139), respiratory tract specimens (27/139), blood and body fluids (28/139), respectively (Supplementary Materials Table S3). Of the total genotypically confirmed PMQR and ESBL cases (N = 139), the most frequently detected PMQR gene was *aac(6′)-lb-cr* 77.7% (108/139) followed by *oqxB* 67.63% (94/139), *oqxA* 62.59% (87/139), *qnrB* 43.17% (60/139), *qnrD* 19.42% (27/139), *qnrS* 18.71% (26/139), *qnrA* 9.35% (13/139), *qepA* 3.6% (5/139), and *qnrC* 2.88% (4/139), respectively (Supplementary Materials Table S3). Among the genotypically confirmed ESBL and PMQR positive *Enterobacterales* isolates that carrying *qnrA* gene (N = 13), majority of the isolates to had co-existence with *bla_TEM_* (13/13) and *bla_CTX-M_* (8/13) gene (Table 4). Similarly, most of the strains carrying *qnrB* gene (N = 60) demonstrated coexistence with *bla_TEM_* (46/60), *bla_CTX-M_* (43/60), and *bla_SHV_* (27/60). In contrast, strains carrying *Aac-ib-cr* gene (N = 108), most of the isolates showed coexistence with *bla_TEM_* (98/108), *bla_CTX-M_* (84/108), *bla_SHV_* (47/108), *bla_PER_* (28/108), and *bla_VEB_* (13/108) (Supplementary Materials Table S3). This coexistence of genes was observed among those isolates that carried *oqxA* and *oqxB* as well. Among the isolates that carrying *oqxA* (N = 87) gene, majority of the isolates were possessing *bla_TEM_* (72/87), *bla_CTX-M_* (66/87) and *bla_SHV_* (39/87), *bla_PER_* (17/87) and *bla_VEB_* (12/87), respectively (Table 4 and Supplementary Materials Table S3). Similarly, isolates that possess *oqxB* genes was carrying *bla_TEM_* (80/94), *bla_CTX-M_* (71/94) and *bla_SHV_* (46/94), *bla_PE_*_R_ (15/94) and *bla_VEB_* (11/94), respectively. It was also noted that *Enterobacterales* isolates that having higher minimum inhibitory concentration (MIC) values for ciprofloxacin (≥16 μg/mL), nalidixic acid (≥64 μg/mL), levofloxacin (≥32 μg/mL),gatifoxacin (≥32 μg/mL) and moxifloxacin (≥32 μg/mL) mainly harboured *Aac-ib-cr, qnrB, oqxA* and *oqxB* genes. Further, the frequency of PMQR genes were high in isolates with higher quinolone MIC than their low MIC counterparts (Supplementary Materials Table S6).

Out of the 60.82% (104/171) of AME genes detected, the distribution of these genes in the *Enterobacterales* (N = 104) is as follows; *K. pneumoniae* 44.23% (46/104), *E. coli* 38.46% (40/104), *P. mirabilis* 2.89% (3/104), *C. freundii* 5.77% (6/104), *K. oxytoca* 3.84% (4/104), *E. cloacae* 2.89% (3/104), *P. vulgaris* 0.96% (1/104), and *M. morganii* 0.96% (1/104), respectively (Table 3). The distribution of different AME genes in various specimens include wound (N = 69), respiratory tract specimens (N = 7), blood and body fluids (N = 28), respectively. Of the total genotypically confirmed ESBL and AME cases (N = 104), the most frequently encountered AME gene was *aac(6′)-Ib* 81.73% (85/104), followed by *aph(3′)-Ia* 46.15% (48/104), *ant(3”)-Ia* 44.23% (46/104), *aac(3)-IIa* 45.19% (47/104), *aph(3”)-Ib* 35.58%, (37/104), *armA* 14.42% (15/104), *ant(4”)-IIa* 12.5% (13/104), *aac(3)-Ib* 10.58%(11/104), *ant(2”)-Ia* 8.65% (9/104), *aac(3)-Ia* 6.73% (7/104), respectively (Table 3). However, *aac(2′)-Ia, aac(6′)-Ia, aac(6′)-Ic* and *aph(3”)-Ia* were not detected in any of the strains analyzed (Supplementary Materials Table S4).

Of the total AME positive Enterobacterale isolates (N = 104) that carried aac(3)-IIa genes (N = 47), majority of the isolate had co-existence with *bla_TEM_* (43/47) and *bla_CTX-M_* (38/47) genes. Similarly, strains carrying *aac(6′)-Ib* gene (N = 85) demonstrated coexistence with *bla_TEM_* (75/85), *bla_CTX-M_* (72/85), and *bla_SHV_* (43/85). However, strains carrying *ant (3”)-Ia* gene (N = 46), majority of isolates showed co-existence with *bla_TEM_* (41/46), *bla_CTX-M_* (35/46) and *bla_SHV_* (23/46). This coexistence of genes were also observed among the isolates that carrying *aph(3”)-Ia* and *aph(3”)-Ib* wherein majority of the strains were equally harbouring both *bla_TEM_* and *bla_CTX-M_* genes (Table 4 and Supplementary Materials Table S4). Further, isolates that showed high MIC values for gentamicin (≥16 μg/mL), tobramycin (≥16 μg/mL), amikacin (≥64 μg/mL) and kanamycin (≥64 μg/mL) mainly harboured *aac(6′)-Ib*, *aph(3”)-Ia*, *aac(3)-IIa, ant(3”)-Ia* genes, respectively (Supplementary Materials Table S7). The rate of incidence of aminoglycoside resistance genes was high in isolates that having higher aminoglycoside MICs as compared to strains with low degree of aminoglycoside MICs (Supplementary Materials Table S7).

Of the 63.74% (108/171) of TMP-SMX resistant ESBL producing *Enterobacterales* isolates, the distribution of TMP-SMX resistance genes are as follows; *K. pneumoniae* 42.59% (46/108), *E. coli* 34.26% (37/108), *P. mirabilis* 5.56% (6/108), *C. freundii* 6.5% (7/108), *K. oxytoca* 5.56% (6/108), *E. cloacae* 4.63% (5/108), *P. vulgaris* 1.86% (2/108), and *M. morganii* 0.92% (1/108), respectively (Table 3). These TMP-SMX resistance genes were distributed among wound (N = 78), respiratory tract specimens (N = 4), blood and body fluids (N = 28), respectively. Of the total genotypically confirmed ESBL and TMP-SMX resistant isolates (N = 108), the most common TMP-SMX resistance gene was *sul1* 100% (108/108), followed by *dfrA* 54.63% (59/108) and *sul2* 15.74% (17/108), respectively (Supplementary Materials Table S5).

Of the total ESBL isolates that carrying sul1 gene (N = 108), majority of the isolates were having co-existence with *bla_TEM_* (95/108), *bla_CTX-M_* (81/108) and *bla_SHV_* (51/108) genes, respectively. Similarly, strains carrying *dfrA1* gene (N = 59) demonstrated coexistence with *bla_TEM_* (53/59) and *bla_CTX-M_* (48/59) (Supplementary Materials Table S5). Further, *Enterobacterales* isolates that showed elevated MICs (4/76 ≥ 16/304 μg/mL) for TMP-SMX mainly harbored *sul1* and *dfrA1* genes. It was also observed that the frequency of TMP-SMX genes was higher in strains with high TMP-SMX MIC values (Supplementary Materials Table S8).

Table 5 summarizes the comparison of origin of strain and type of resistance genes detected. The data analysis shows no direct correlation exists between origin of strain (wound, respiratory tract specimens, blood and body fluids) and the type of resistance genes (ESBL, PMQR, AME, and TMP-SMX resistance genes) detected. The number of types of resistance genes detected in wound specimens were significantly (*p* < 0.05) different from that found in the respiratory tract specimens (Table 5).

## 3. Discussion

The current study investigated the frequency of non-β-lactam antibiotic resistance associated genes among ESBL producing *Enterobacterales*. It was observed that the prevalence of ESBL genes detected in the current study (particularly among *E. coli* and *K. pneumoniae)* were comparable with earlier studies [2,22]. The most commonly encountered ESBL gene in this study was *bla_TEM_* (84.79%) followed by *bla_CTX-M_* (73.68%), *bla_SHV_* (43.86%), *bla_PE_*_R_ (18.71%) and *bla_SHV_* (9.94%), respectively. These findings were in accordance with an earlier study wherein most prevalent ESBL gene found was *bla_TEM_* (73%) followed by *bla_CTX-M_* (25–100%) and *bla_SHV_* (23%) [2]. The low prevalence of *bla_PER_* and *bla_VEB_* in the present study were comparable with the data reported by Khurana et al. [2]. Further, it was observed that most of the ESBL producing organisms were resistant to fluoroquinolones, aminoglycosides and TMP-SMX, respectively. This may be possibly due to the co-existence of PMQR, AME and TMP-SMX resistance genes in the same plasmids that also code for ESBL proteins [22].

In the present study, PCRs detected presence of PMQR genes in 81.29% (N = 139) of genotypically confirmed ESBL isolates (N = 171), indicating the presence of high frequency of PMQR genes among ESBL strains. Interestingly, *K. pneumoniae* (44.6%) was having higher number of PMQR genes detected followed by *E. coli* (31.65%). This observation is in accordance with an earlier study wherein, PMQR genes were more frequently encountered among *E. coli, Klebsiella species,* and *Enterobacter species* [3]. However, the frequency of occurrence of PMQR genes were low among *Enterobacter cloacae* in this study. This is probably due to the difference in the geographical location as the earlier study was conducted in the southern part of India (where the usage of antibiotics is different). Further, the widespread antibiotic resistance prevalent in India may be attributed to readily availability of antibiotics across the pharmacy counters. This could play a major role in increased distribution of antibiotic-resistance genes throughout the population. This study included isolates that obtained from wound, respiratory tract, body fluid and blood specimens while other studies were performed mostly using isolates that are collected from urine [4,9,20]. Of the total genotypically confirmed ESBL cases (N = 171), the most frequent PMQR gene detected was *aac(6′)-lb-cr* (77.7%) which is in agreement with an earlier report wherein the prevalence rate was found to be 64.5% [3]. In this study, relatively higher prevalence of *qnrB* (43.17%) was observed, which was in accordance with Yang et.al observation (prevalence rate ~50%) [13]. Previous studies also reported the low prevalence of *qnrD, qnrS, qnrA*, and *qnrC* [4,5,22,23]. It was observed that the distribution of efflux pumps genes among ESBL producing *Enterobacterales* isolates were found to be *oqxB* (67.63%), *oqxA* (62.59%) and *qepA* (3.6%), respectively. However, this efflux pump mediated drug resistance mechanism of bacteria can be subdued using various efflux inhibitory molecules [24], for instance, the susceptibility of antibiotics against multidrug-resistant bacteria (that developed exclusively due to efflux pump mechanisms) can be enhanced in presence of efflux pump inhibitor such as omeprazole [25]. Further, the genome sequencing analysis of multidrug-resistant strains that might reveal the potential genes that are associated with multidrug efflux pumps and once the genes and gene products are identified, the molecular docking studies that may further help in developing appropriate efflux pump inhibitors. These efflux pump inhibitors can be incorporated with antibiotic molecules in order to overcome the efflux pump mediated drug resistance [26]. Further, to our knowledge, this is the first study that investigated the prevalence of PMQR among *Enterobacterales in* North India. However, the *oqxA* and *oqxB* prevalence rates were comparable with earlier reports wherein the prevalence rates were found to be 88% and 30% for *oqxA* and *oqxB* genes, respectively [27]. Further, the differences in the prevalence of *oqxA* and *oqxB* genes may be attributed to the geographical distribution and type of isolates studied, as most of the studies were conducted on *E. coli* and K. *pneumonia* isolates [5,19]. The low prevalence of *qepA* observed (3.6%) was similar with previous data (2%) in the literature [13,28]. This low prevalence of *qepA* in the current study may also indicate low incidence of *qepA gene* among different strains of *Enterobacterales* across the world [5]. In this study, the presence of PMQR genes were associated with ESBL genes, possibly due to the common carriage on the same plasmids [23]. The isolates that carrying a minimum of two β-lactamases coding genes (particularly, *bla_TEM_* and *bla_CTX-M_*) were more likely to carry *aac(6′)-Ib-cr* and *qnrB* genes. The genes that code for both ESBL and PMQR proteins are usually located on same plasmids and consequently that may have higher chances of transfer among the members of *Enterobacterales*. Therefore, it is very pertinent to comprehend the drug resistance mechanisms prevalent among the members of medically important bacteria as it may be a major concern for patient safety and in determination of therapeutic strategies.

Genes encoding AMEs are prevalent in various groups of bacteria [10,29,30,31,32]. In this study, prevalence of AME genes were found to be 60.82% among ESBL producing strains of *Enterobacterales*. This relatively higher prevalence rate of AMEs in ESBL producing strains may be due to co-existence of genes encoding ESBLs and AMEs in Gram-negative bacteria [9]. In this study, AMEs coding genes were most frequently isolated from *K. pneumoniae* (44.23%) and *E. coli* (38.46%). This was in accordance with an earlier study conducted by Haidar et al., wherein a higher prevalence of AME genes were reported among *K. pneumoniae* [9]. In the present study, the most frequently encountered AME gene was *aac(6′)-Ib* (81.73%) which is in agreement with the earlier studies (prevalence was 73%) [9]. The predominance and coexistence of *aac(6′)-Ib* with other AME genes observed may be attributed to the fact that the gene coding for *aac(6′)-Ib* enzyme is frequently located within class I integrons. Further, it is known that the gene cassettes that carry other genes coding for AMEs can be easily incorporated into class I integrons resulting in the development of resistance to currently used aminoglycosides [10]. The other prevalent genes were *aph(3′)-Ia* (46.15%), followed by *ant(3”)-Ia* (44.23%), *aac(3)-IIa* (45.19%), and *aph(3”)-Ib* (35.58%). The higher prevalence of *aph(3′)-Ia* and *aph(3”)-Ib* is alarming as this type of resistance may usually produce high level of aminoglycoside resistance. The prevalence of other AME genes were found to be relatively low, which is comparable with earlier studies [9,23].

The TMP-SMX resistance genes such as *sul1*, *sul2,* or *dfrA* genes are likely to be present either on chromosome or on plasmids [33,34]. In the present study, TMP-SMX resistance genes were obtained from 63.74% of ESBL producing isolates. This comparatively low detection rate of these resistance genes may be attributed to the presence of alternative resistance mechanisms prevailing in TMP-SMX resistant isolates [33]. However, additional investigations are required to explore the genetic basis of TMP-SMX resistance mechanisms that prevailing in various strains of *Enterobacterales.* Further, among the genotypically confirmed TMP-SMX resistant strains (N = 108), 42.59% of *K. pneumonia* isolates were carrying TMP-SMX resistance genes, followed by *E. coli* (34.26%). However, due to the paucity of literatures, the comparison with earlier studies could not performed. To our knowledge, this new study report, the prevalence of TMP-SMX resistance genes among various clinical strains of ESBL producing *Enterobacterales*. A higher prevalence of *sul1* (100%) followed by *dfrA* (54.63%) and *sul2* (15.74%), genes were noted in this study. This higher prevalence of *sul1* gene may be attributed to the fact that the *sul*1 genes are usually located within class I integrons and this particular characteristic (which is a horizontally transferable genetic element) might have further helped in its wide distribution [35]. The comparatively higher prevalence of *dfrA*1 (33.91%) gene in the present study was in agreement with an earlier study wherein in the prevalence rate was also found to be high [22]. *Enterobacterales* isolates that showed elevated MICs for TMP-SMX mainly harbored *sul1* and *dfrA1* genes indicating the likelihood of these genes in imparting resistance to TMP-SMX. However, no genes were detected from number of isolates that were having higher MIC values, suggesting the existence of alternative pathways of resistance in TMP-SMX resistant isolates.

## 4. Materials and Methods

### 4.1. Study Setting and Clinical Specimens

Between July 2018 and June 2019, a total of 2134 clinical samples (wound, respiratory tract specimens, blood and body fluids) received in the Microbiology laboratory, Government Medical College and Hospital, Badaun, India were analyzed for ESBL producing strains of *Enterobacterales*. This hospital laboratory receives samples from two civil hospitals and three primary health care centers that are attached to it. Among the total samples analyzed (N = 2134), a total of 186 non-repetitive phenotypically confirmed ESBL producing *Enterobacterales* isolates (one organism per patient was included to avoid duplication) were obtained. All the isolates were identified by manual API^®^ system (BioMérieux, Durham, NC, USA) and the results interpreted as recommended by the manufacturer. The strains included were *K. pneumoniae* (N = 74), *E. coli* (N = 58), *P. mirabilis* (N = 15), *C. freundii* (N = 13), *K. oxytoca* (N = 11), *E. cloacae* (N = 9), *P. vulgaris* (N = 3) and *M. morganii* (N = 3). All the clinical isolates were identified by standard laboratory procedure [11]. All the bacterial isolates were stored at −80 °C in glycerol for future use.

### 4.2. Antimicrobial Susceptibility Testing and MIC Determination

The antibiotic susceptibility testing was conducted by modified Kirby Bauer disc diffusion method as recommended by the Clinical Laboratory Standards Institute (CLSI) [10]. The antibiotic discs (HiMedia, Mumbai, India) tested include; ampicillin (10 μg), cefazolin (30 μg), amoxicillin -clavulanic acid (20 μg + 10 μg), cefotaxime (30 μg), cefepime (30 μg), ceftazidime (30 μg), ceftriaxone (30 μg), cefoxitin (10 μg), cefpodoxime (30 μg), cefuroxime (30 μg), ceftizoxime (30 μg), imipenem (10 μg), meropenem (10 μg), aztreonam (30 μg), gentamicin (10 μg), tobramycin (10 μg), amikacin (30 μg), kanamycin (30 μg), ciprofloxacin (5 μg), levofloxacin (5 μg), gatifloxacin (5 μg), nalidixic acid (30 μg), moxifloxacin (5 μg), and TMP-SMX (1.25/23.75 μg). However, some of the antibiotics were not tested against the following organisms since these organism’s possess intrinsic resistance, more specifically, (a) *K. pneumoniae*, *C. freundii*, *K. oxytoca*, *E. cloacae*, *P. vulgaris*, and *M. morganii* against Ampicillin (b) *C. freundii, E. cloacae,* and *M. morganii* against amoxicillin- clavulanic acid (c) *C. freundii*, and *E. cloacae* against cefoxitin (d) *P. vulgaris*, and *M. morganii* against cefpodoxime, respectively [36]. Quality control strains used for antimicrobial susceptibility testing include *E. coli* (ATCC 25922) and Pseudomonas aeruginosa (ATCC 27853).

The MIC of fluoroquinolones (MIC determined for selected antibiotics that include; ciprofloxacin, levofloxacin, nalidixic acid, gatifloxacin, and moxifloxacin), aminoglycosides (MIC calculated for; gentamycin, tobramycin, amikacin and kanamycin) and TMP-SMX were determined by broth micro dilution method and the results were interpreted in accordance with the CLSI guidelines [36]. The MICs were calculated to determine the association between presence of antibiotic resistance genes and concentration of antibiotic tested.

### 4.3. Phenotypic Detection of ESBL

ESBL activity was initially screened using disk diffusion method as recommended by CLSI [36]. Isolates that showed decreased zone of inhibition to one or more of the following antibiotic: ≤25 mm with ceftriaxone (30 μg), ≤27 mm with cefotaxime (30 μg), ≤22 mm with ceftazidime (30 μg), ≤17 mm with cefpodoxime (10 μg), and ≤27 mm with aztreonam (30 μg) were considered as ESBL producers (for *E. coli, K. pneumoniae,* and *K oxytoca*). However, zone size with ≤22 mm cefpodoxime (10 μg), ≤22 mm with ceftazidime (30 μg), ≤27 mm with cefotaxime, indicated ESBL production for *P. mirabilis* [36].

The double-disk synergy diffusion test (phenotypic confirmatory test for ESBL) was carried out as per CLSI recommendations. Briefly, ceftazidime (30 μg) and cefotaxime (30 μg) alone and in combination with clavulanic acid (10 μg; Himedia, Mumbai, India) were used. The ESBL production is confirmed when there is an increase of zone diameter (≥5 mm) around disk with antibiotic-clavulanic acid combination [36].

The disc approximation test was used to confirm the ESBL production in *E. cloacae, C. freundii, P. vulgaris,* and *M. morganii* strains. This test was performed as disc diffusion assay on Mueller-Hinton agar (MHA). Briefly, antibiotic discs containing aztreonam (30 μg), ceftazidime (30 μg), ceftriaxone (30 μg), and cefotaxime (30 μg) were kept 30 mm apart (center to center) around amoxicillin-clavulanic acid (20 μg + 10 μg) disc on MHA plate inoculated with the organism to be tested. The MHA plates were incubated at 37 °C for 24 h. An increased zone of inhibition of any of the test antibiotic towards amoxicillin-clavulanic acid was considered as ESBL production [37]. The control strains used were *E. coli* ATCC 25922 (non-beta-lactamases producer) and *K. pneumoniae* ATCC 700603 (ESBL producer), respectively.

### 4.4. PCR Analysis and Sequencing

#### 4.4.1. Extraction of Bacterial DNA

Bacterial DNA was obtained from the phenotypically confirmed ESBL strains of *Enterobacterales* using QIAamp DNA Mini Kit (Qiagen, Hilden, Germany). The DNA samples obtained by this procedure were segregated into two aliquots; first aliquot was used as template for the subsequent PCR reactions and the second aliquot was stored at −80 °C (Jindal Ultra Freezer (SMI-165E), Ghaziabad, India) for future use.

#### 4.4.2. Molecular Detection of ESBL, PMQR, AME and TMP-SMX Resistance Genes

All the phenotypically confirmed ESBL producing *Enterobacterales* isolates were subjected to molecular characterization of the relevant encoding genes such as *bla_SHV_, bla_CTX-M_, bla_TEM_, bla_PER_*, and *bla_VEB_* by PCR using primers and PCR conditions shown in Table 1 [14,15].

ESBL producing *Enterobacterales* isolates that resistant to fluoroquinolones were subjected to PCRs for detection of (i) qnr proteins coding genes such as *qnrA, qnrB, qnrC, qnrD*, and *qnrS* (ii) quinolones modifying aminoglycoside acetyltransferase coding genes *(acc(6′)-Ib-cr*) and (iii) plasmid-mediated quinolone efflux pump protein encoding genes (*qepA, oqxA and oqxB*) using primers and PCR conditions depicted in Table 1 [4,5,16,17,18,19].

ESBL producing aminoglycosides resistant strains of *Enterobacterales* were screened for AMEs coding genes *aac(2′)-Ia, aac(3)-Ia, aac(3)-Ib, aac(3)-IIa, aac(6′)-Ia, aac(6′)-Ib,aac(6′)-Ic,ant(2”)-Ia, ant(3”)-Ia, ant(4”)-IIa, aph(3′)-Ia, aph(3”)-Ia, aph(3”)-Ib, armA* using primers described in Table 1 [20,21].

All isolates phenotypically resistant to TMP-SMX were subjected to PCR for detection of *sul1, sul2* and *dfrA* genes using primers and PCR conditions shown in Table 1 [22,38]. Briefly, 2 µL (~500 ng) of purified DNA was subjected to each multiplex PCR in a 100 µL reaction mixture containing 1 × PCR buffer (10 Tris-HCl pH 8.8, (NH_4_)_2_SO_4_, 3 mM MgCl_2_, 0.2% Tween 20), 200 mM of each dNTPs, 0.5 µM of each primer, and 2.0 units of AURA Taq DNA polymerase. Amplification was carried out as follows: initial denaturation at 95 °C for 5 min; 30 cycles of denaturation at 95 °C for 30 s, annealing at 50–62 °C for 30 s, extension/elongation at 72 °C for 45 s; and a final elongation step at 72 °C for 5 min. PCR-generated products were detected by electrophoresis of 7 μL of each amplification mixture in 2% agarose gels in 1% Tris Borate-EDTA buffer and 0.5 µg/mL ethidium bromide.

To identify the ESBL, PMQR, AME, and TMP-SMX resistance genes detected in the PCR assays, automated DNA sequencing of the amplicons were conducted. More specifically, multiplex PCR-generated products were separated in 2% low melting agarose gel with 1% Tris-acetate-EDTA buffer. The PCR products were excised from the agarose gel and purified using the QIAquick PCR purification kit (Qiagen, Hilden, Germany) as recommended by the manufacturer. The nucleotide sequencing of amplicons was conducted using an ABI 3730xl DNA Analyzer (Applied Biosystems, Branch burg, NJ, USA). Basic Local Alignment Search Tool (BLAST) program was used to compare each ESBL, PMQR, AME, and TMP-SMX resistance gene sequences against those available in gene bank at the National Center of Biotechnology Information database.

### 4.5. Statistical Analysis

Chi-squared test was used to compare the association between the origin of strain and type of resistance genes detected. The null hypothesis will be accepted if the presence of genes in all the groups (wound, respiratory tract specimens, and blood body fluids) were similar. Dunn’s multiple comparisons test was performed to compare the differences in the number of resistance genes obtained between two categories of samples. All statistical analyses were performed using Graph pad Prism (version 6, Graph-Pad Software, Inc., La Jolla, CA, USA). The statistical difference values showing *p* < 0.05 were considered as significant.

## 5. Conclusions

In summary, this is the first study that investigated the occurrence of genetic determinants prevailing in ESBL producing *Enterobacterales* and the association of these genetic determinants with PMQR, AME, and TMP-SMX resistance genes in the north India. The current study demonstrated widespread occurrence of PMQR, AME, and TMP-SMX drug resistant genetic determinants in the ESBL producing *Enterobacterales* strains. Screening of PMQR genetic elements in ESBL producing *Enterobacterales* strains revealed high prevalence of both *aac(6′)-Ib-cr* and *qnrB.* However, molecular analysis of AMEs producing *Enterobacterales* strains showed high prevalence of *aac(6′)-Ib* followed by *aph (3′)-Ia.* On examination of TMP-SMX resistant strains, *sul1* was found to be the most frequently encountered gene followed by *dfrA*. The association of ESBL-producing genes with the PMQR, AMEs, and TMP-SMX resistance genes may potentially aid in transfer of drug resistance determinants among these strains. Therefore, a complete understanding of PMQRs, AMEs, and other drug resistance mechanisms will help in determining rationale of treatment and infection control measures in hospital settings.

## Figures and Tables

**Table 1:** Primers used for PCR and sequencing of drug resistance-associated genes from *Enterobacterales*.

Target Gene	Primer Name	Primer Sequence (5′–3′)	Annealing Temperature (°C)	Amplicon/Product Size (bp)	References
ESBL genes	*TEM*	F AGATCAGTTGGGTGCACGAG	52 °C	750	[14]
		R TGCTTAATCAGTGAGGCACC			
	*SHV*	F GGGAAACGGAACTGAATGAG	55 °C	380	[14]
		R TTAGCGTTGCCAGTGCTCG			
	*CTX-M1*	F TTAGGAARTGTGCCGCTGYA	60 °C	688	[15]
		R CGATATCGTTGGTGGTRCCAT			
	*PER*	F GCTCCGATAATGAAAGCGT	60 °C	520	[15]
		R TTCGGCTTGACTCGGCTGA			
	*VEB*	F CATTTCCCGATGCAAAGCGT	60 °C	648	[15]
		R CGAAGTTTCTTTGGACTCTG			
PMQR gene	*qnrA*	F AGAGGATTTCTCACGCCAGG	57 °C	630	[4]
		R GCAGCACTATKACTCCCAAGG			
	*qnrB*	F GGMATHGAAATTCGCCACTG	57 °C	264	[5]
		R TTTGCYGYYCGCCAGTCGAA			
	*qnrC*	F GGGTTGTACATTTATTGAATC	57 °C	447	[16]
		R TCCACTTTACGAGGTTCT			
	*qnrD*	F CGAGATCAATTTACGGGGAATA	57 °C	582	[17]
		R AACAAGCTGAAGCGCCTG			
	*qnrS*	F GCAAGTTCATTGAACAGGGT	57 °C	428	[15]
		R TCTAAACCGTCGAGTTCGGCG			
	*aac(6′)-Ib-cr*	F TTGGAAGCGGGGACGGAM	52 °C	260	[18]
		R ACACGGCTGGACCATA			
	*oqxA*	F GACAGCGTCGCACAGAATG	62 °C	339	[4]
		R GGAGACGAGGTTGGTATGGA			
	*oqxB*	F CGAAGAAAGACCTCCCTACCC	62 °C	240	[4]
		R CGCCGCCAATGAGATACA			
	*qepA*	F GCAGGTCCAGCAGCGGGTAG	62 °C	218	[19]
		R CTTCCTGCCCGAGTATCGTG			
AME genes	*aac(2′)-Ia,*	F AGAAGCGCTTTACGATTTATTA	55 °C	406	[20]
		R GACTCCGCCTTCTTCTTCAA	55 °C		
	*aac(3)-Ia*	F GCAGTCGCCCTAAAACAAA	55 °C	441	[20]
		R CACTTCTTCCCGTATGCCCAACTT			
	*aac(3)-Ib*	F GCAGTCGCCCTAAAACAAA	55 °C	417	[20]
		R GGATCGTCACCGTAGTCTGC			
	*aac(3)-IIa*	F GGCAATAACGGAGGCGCTTCAAAA	55 °C	563	[20]
		R TTCCAGGCATCGGCATCTCATACG			
	*aac(6′)-Ia*	F ATGAATTATCAAATTGTG	55 °C	558	[20]
		R TTACTCTTTGATTAAACT			
	*aac(6′)-Ib*	F CAAAGTTAGGCATCACA	55 °C	540	[20]
		R ACCTGTACAGGATGGAC			
	*aac(6′)-Ic*	F CTACGATTACGTCAACGGCTGC	55 °C	130	[20]
		R TTGCTTCGCCCACTCCTGCACC			
	*ant(2″)-Ia*	F ACGCCGTGGGTCGATGTTTGATGT	55 °C	572	[20]
		R CTTTTCCGCCCCGAGTGAGGTG			
	*ant(3″)-Ia*	F TCGACTCAACTATCAGAGG	55 °C	245	[20]
		R ACAATCGTGACTTCTACAGCG			
	*ant(4″)-IIa*	F CCGGGGCGAGGCGAGTGC	55 °C	423	[20]
		R TACGTGGGCGGATTGATGGGAACC			
	*aph(3′)-Ia*	F CGAGCATCAAATGAAACTGC	55 °C	625	[20]
		R GCGTTGCCAATGATGTTACAG			
	*aph(3″)-Ia*	F CGGCGTGGGCGGCGACTG	55 °C	557	[20]
		R CCGGATGGAGGACGATGTTGG			
	*aph(3″)-Ib*	F GTGGCTTGCCCCGAGGTCATCA	55 °C	612	[20]
		R CCAAGTCAGAGGGTCCAATC			
	*armA*	F ATTTTAGATTTTGGTTGTGGC	54.5 °C	101	[21]
		R ATCTCAGCTCTATCAATATCG			
		R TACGTGGGCGGATTGATGGGAACC			
TMP-SMX resistance genes	*sul1*	F CGGCGTGGGCTACCTGAACG	55 °C	432	[22]
		R GCCGATCGCGTGAAGTTCCG			
	*sul2*	F GCGCTCAAGGCAGATGGCATT	53 °C	293	[22]
		R GCGTTTGATACCGGCACCCGT			
	*dfrA1*	F TGGAGTTATCGGGAATGGC	34 °C	334	[22]
		R AACATCACCTTCCGGCTCG			

**Table 2:** Antibiotic susceptibility pattern of the *Enterobacterales* isolates obtained from wound, respiratory tract and blood and body fluid specimens.

Antibiotics	Organisms							
	*Escherichia coli*, N = 58 (%)	*Klebsiella Pneumoniae*, N = 74 (%)	*Proteus mirabilis*, N = 15 (%)	*Citrobacter freundii*, N = 13 (%)	*Klebsiella oxytoca*, N = 11 (%)	*Enterobacter cloacae*, N = 9 (%)	*Proteus vulgaris*, N = 3 (%)	*Morganella morganii*, N = 3 (%)
Ampicillin	55 (94.83)	-	15 (100)	-	-	-	-	-
Cefazolin	49 (84.48)	69 (93.24)	13 (86.67)	12 (92.31)	10 (90.91)	7 (77.78)	3 (100)	3 (100)
Amoxicillin-clavulanic acid	38 (65.52)	64 (86.49)	12 (80)	-	8 (72.73)	-	-	-
Cefotaxime	48 (82.76)	63 (85.14)	9 (60)	13 (100)	8 (72.73)	9 (100)	2 (66.67)	2 (66.67)
Cefepime	46 (79.31)	41 (55.41)	10 (66.67)	11 (84.62)	9 (81.82)	5 (55.56)	1 (33.33)	2 (66.67)
Ceftazidime	52 (89.66)	66 (89.19)	13 (86.67)	11 (84.62)	11 (100)	9 (100)	2 (66.67)	3 (100)
Ceftriaxone	50 (86.21)	71 (95.95)	8 (53.33)	13 (100)	11 (100)	7 (77.78)	1 (33.33)	2 (66.67)
Cefoxitin	41 (70.69)	64 (86.49)	11 (73.33)	-	7 (63.64)	-	2 (66.67)	1 (33.33)
Cefpodoxime	52 (89.66)	66 (89.19)	7 (46.67)	10 (76.92)	11 (100)	9 (100)	-	-
Cefuroxime	50 (86.21)	69 (93.24)	9 (60)	11 (84.62)	10 (90.91)	7 (77.78)	2 (66.67)	2 (66.67)
Ceftizoxime	47 (81.03)	67 (90.54)	10 (66.67)	10 (76.92)	8 (72.73)	9 (100)	2 (66.67)	2 (66.67)
Imipenem	22 (37.93)	30 (40.54)	12 (80)	7 (53.85)	6 (54.55)	3 (33.33)	0	0
Meropenem	27 (46.55)	32 (43.24)	11 (73.33)	7 (53.85)	6 (54.55)	3 (33.33)	1 (33.33)	0
Aztreonam	54 (93.1)	68 (91.89)	12 (80)	10 (76.92)	11 (100)	9 (100)	1 (33.33)	2 (66.67)
Gentamicin	40 (68.97)	46 (62.16)	7 (46.67)	5 (38.46)	4 (36.36)	8 (88.89)	2 (66.67)	2 (66.67)
Tobramycin	45 (77.59)	50 (67.57)	5 (33.33)	7 (53.85)	6 (54.55)	7 (77.78)	-	1 (33.33)
Amikacin	32 (55.17)	39 (52.7)	3 (20)	3 (23.08)	5 (45.45)	6 (66.67)	1 (33.33)	-
Kanamycin	40 (68.97)	36 (48.65)	8 (53.33)	6 (46.15)	6 (54.55)	9 (100)	2 (66.67)	1 (33.33)
Ciprofloxacin	45 (77.59)	65 (87.84)	11 (73.33)	8 (61.54)	6 (54.55)	4 (44.44)	1 (33.33)	2 (66.67)
Levofloxacin	32 (55.17)	56 (75.68)	11 (73.33)	7 (53.85)	8 (72.73)	6 (66.67)	2 (66.67)	2 (66.67)
Nalidixic acid	40 (68.97)	64 (86.49)	12 (80)	5 (38.46)	8 (72.73)	5 (55.56)	1 (33.33)	2(66.67)
Gatifloxacin	46 (79.31)	62 (83.78)	10 (66.67)	6 (46.15)	7 (63.64)	4 (44.44)	2 (66.67)	2 (66.67)
Moxifloxacin	42 (72.41)	59 (79.73)	12 (80)	7 (53.85)	7 (63.64)	3 (33.33)	1 (33.33)	2 (66.67)
Trimethoprim- sulfamethoxazole	57 (98.28	65 (87.84)	15 (100)	11 (84.62)	9 (81.82)	9 (100)	2 (66.67)	3 (100)

**Table 3:** Distribution of Extended-spectrum beta-lactamases (ESBL) and non-beta-lactamase encoding genes in genotypically confirmed strains of ESBL producing *Enterobacterales*.

Type of Resistance	Organisms							
	*Escherichia coli*, N = 53 (%)	*Klebsiella pneumoniae*, N = 69 (%)	*Proteus mirabilis*, N = 13 (%)	*Citrobacter freundii*, N = 12 (%)	*Klebsiella oxytoca*, N = 9 (%)	*Enterobacter cloacae*, N = 9 (%)	*Proteus vulgaris*, N = 3 (%)	*Morganella morganii*, N = 3 (%)
**ESBL**								
*TEM*	46 (86.8)	58 (84.06)	12 92.31)	9 (75)	7 (77.78)	7 (77.78)	3 (100)	3 (100)
*CTX-M*	39 (73.6)	61 (88.41)	8 (61.54)	8 (66.67)	6 (66.67)	4 (44.44)	ND	ND
*SHV*	29 (54.7)	30 (43.48)	2 (15.39)	4 (33.33)	7 (77.78)	3 (33.33)	ND	ND
*PER*	8 (15.1)	13 (18.84)	3 (23.08)	3 (25)	2 (22.22)	3 (33.33)	1 (33.33)	ND
*VEB*	1 (1.89)	5 (7.25)	8 (61.54)	ND	1 (11.11)	2 (22.22)	ND	ND
**PMQR**								
*qnr A*	3 (5.66)	ND	8 (61.54)	ND	ND	ND	2 (66.67)	ND
*qnrB*	12 (22.6)	36 (52.17)	ND	6 (50)	5 (55.56)	ND	ND	1 (33.33)
*qnrC*	4 (7.55)	ND	ND	ND	ND	ND	ND	ND
*qnrD*	7 (13.2)	17 (24.64)	ND	ND	3 (33.33)	ND	ND	ND
*qnrS*	11 (20.8)	3 (4.35)	9 (69.23)	ND	ND	3 (33.33)	ND	ND
*Aac-ib-cr*	32 (60.4)	51 (73.91)	7 (53.85)	7 (58.33)	6 (66.67)	2 (22.22)	3 (100)	ND
*oqxA*	14 (26.4)	58 (84.06)	9 (69.23)	ND	4 (44.44)	2 (22.22)	ND	ND
*oqxB*	21 (39.6)	62 (89.86)	8 (61.54)	ND	3 (33.33)	ND	ND	ND
*qepA*	5 (9.43)	ND	ND	ND	ND	ND	ND	ND
**AME**								
*aac(3)-Ib*	2 (3.77)	4 (5.8)	ND	3 (25)	1 (11.11)	1 (11.11)	ND	ND
*aac(3)-Ia*	6 (11.3)	ND	ND	1 (8.33)	ND	ND	ND	ND
*aac(3)-IIa*	18 (34)	19 (27.54)	1(7.69)	3 (25)	2 (22.22)	3 (33.33)	ND	1 (33.33)
*aac(6′)-Ib*	28 (52.8)	46 (66.67)	3 (23.08)	2 (16.67)	4 (44.44)	1 (11.11)	ND	1 (33.33)
*ant(2”)-Ia*	0	6 (8.7)	ND	ND	1 (11.11)	2 (22.22)	ND	ND
*ant(3”)-Ia*	22 (41.5)	17 (24.64)	2 (15.39)	3 (25)	ND	1 (11.11)	1 (33.33)	ND
*ant(4”)-IIa*	8 (15.1)	4(5.797)	1 (7.69)	ND	ND	ND	ND	ND
*aph(3′)-Ia*	16 (30.2)	30 (43.48)	1 (7.69)	ND	1 (11.11)	ND	ND	ND
*aph(3”)-Ib*	20 (37.7)	15 (21.74)	1 (7.69)	ND	2 (22.22)	2 (22.22)	ND	ND
*armA*	5 (9.43)	10 (14.49)	ND	ND	ND	ND	ND	ND
**TMP-SMX**								
*sul1*	35 (66)	46 (66.67)	6 (46.15)	7 (58.33)	6 (66.67)	5 (55.56)	2 (66.67)	1 (33.33)
*sul2*	9 (17)	6 (8.7)	ND	ND	1 (11.11)	1 (11.11)	ND	ND
*dfrA1*	20 (37.7)	28 (40.58)	2 (15.39)	4 (33.33)	1 (11.11)	2 (22.22)	1 (33.33)	1 (33.33)

ND: Not detected.

**Table 4:** Coexistence of ESBL with PMQR, AME and TMP-SMX genes in *Enterobacterales*.

Name of the Gene	Name of the Organism	Total Number of Isolates	ESBL Genes				
PMQR			*TEM*	*CTX-M*	*SHV*	*PER*	*VEB*
*qnr A*	*Escherichia coli*	9	4	3	4	1	ND
	*Proteus mirabilis*	8	7	4	1	2	4
	*Proteus vulgaris*	2	2	ND	ND	1	ND
*qnrB*	*Escherichia coli*	12	7	5	8	ND	ND
	*Klebsiella pneumonia*	36	30	33	15	5	3
	*Citrobacter freundii*	6	5	3	1	1	ND
	*Klebsiella oxytoca*	5	4	2	3	1	1
	*Morganella morganii*	1	1	ND	ND	ND	ND
*qnrC*	*Escherichia coli*	4	4	2	ND	ND	ND
*qnrD*	*Escherichia coli*	7	7	7	4	2	ND
	*Klebsiella pneumonia*	17	15	13	12	7	2
	*Klebsiella oxytoca*	3	3	ND	1	ND	1
*qnrS*	*Escherichia coli*	11	9	11	1	3	1
	*Klebsiella pneumonia*	3	3	ND	2	1	1
	*Proteus mirabilis*	9	9	4	2	2	6
	*Enterobacter cloacae*	3	3	1	ND	1	1
*Aac-ib-cr*	*Escherichia coli*	32	30	29	9	8	1
	*Klebsiella pneumonia*	51	46	43	30	13	5
	*Proteus mirabilis*	7	6	3	1	2	5
	*Citrobacter freundii*	7	5	4	2	1	0
	*Klebsiella oxytoca*	6	5	4	5	2	1
	*Enterobacter cloacae*	2	2	1	ND	1	1
	*Proteus vulgaris*	3	3	ND	ND	1	ND
*oqxA*	*Escherichia coli*	14	10	9	6	ND	ND
	*Klebsiella pneumonia*	58	49	50	30	13	5
	*Proteus mirabilis*	9	8	5	1	2	5
	*Klebsiella oxytoca*	4	3	1	2	1	1
	*Enterobacter cloacae*	2	2	1	ND	1	1
*oqxB*	*Escherichia coli*	21	16	13	13	ND	ND
	*Klebsiella pneumonia*	62	53	54	30	13	5
	*Proteus mirabilis*	8	8	4	2	2	5
	*Klebsiella oxytoca*	3	3	ND	1	ND	1
*qepA*	*Escherichia coli*	5	3	1	4	ND	ND
**AME**							
*aac(3)-Ib*	*Escherichia coli*	2	2	2	ND	ND	ND
	*Klebsiella pneumonia*	4	4	1	2	2	1
	*Citrobacter freundii*	3	1	3	2	ND	ND
	*Klebsiella oxytoca*	1	1	ND	ND	ND	ND
	*Enterobacter cloacae*	1	1	ND	ND	1	ND
*aac(3)-Ia*	*Escherichia coli*	6	6	6	4	3	ND
	*Citrobacter freundii*	1	ND	1	1	ND	ND
*aac(3)-IIa*	*Escherichia coli*	18	16	18	4	4	1
	*Klebsiella pneumonia*	19	17	18	13	5	ND
	*Proteus mirabilis*	1	1	1	1	ND	1
	*Citrobacter freundii*	3	3	1	ND	1	ND
	*Klebsiella oxytoca*	2	2	ND	ND	ND	1
	*Enterobacter cloacae*	3	3	1	ND	1	1
	*Morganella morganii*	1	1	ND	ND	ND	ND
*aac(6′)-Ib*	*Escherichia coli*	28	26	28	8	8	1
	*Klebsiella pneumonia*	46	41	38	30	13	5
	*Proteus mirabilis*	3	3	2	1	1	2
	*Citrobacter freundii*	2	ND	2	2	ND	ND
*aac(6′)-Ib*	*Klebsiella oxytoca*	4	3	1	2	1	1
	*Enterobacter cloacae*	1	1	ND	ND	ND	1
	*Morganella morganii*	1	1	ND	ND	ND	ND
*ant(2”)-Ia*	*Klebsiella pneumonia*	6	4	6	6	2	ND
	*Klebsiella oxytoca*	1	1	ND	ND	ND	ND
	*Enterobacter cloacae*	2	2	1	ND	1	1
*ant(3”)-Ia*	*Escherichia coli*	22	19	17	9	3	ND
	*Klebsiella pneumonia*	17	15	15	13	8	ND
	*Proteus mirabilis*	2	2	1	1	1	2
	*Citrobacter freundii*	3	3	1	ND	1	ND
	*Enterobacter cloacae*	1	1	1	ND	1	ND
	*Proteus vulgaris*	1	1	ND	ND	1	ND
*ant(4”)-IIa*	*Escherichia coli*	8	8	8	3	1	ND
	*Klebsiella pneumonia*	4	4	4	2	ND	ND
	*Proteus mirabilis*	1	1	ND	ND	1	1
*aph(3′)-Ia*	*Escherichia coli*	16	16	16	8	5	ND
	*Klebsiella pneumonia*	30	27	25	22	11	1
	*Proteus mirabilis*	1	1	ND	ND	1	1
	*Klebsiella oxytoca*	1	1	ND	ND	ND	ND
*aph(3”)-Ib*	*Escherichia coli*	20	17	15	9	3	ND
	*Klebsiella pneumonia*	15	15	14	10	5	ND
	*Proteus mirabilis*	1	1	ND	ND	1	1
	*Klebsiella oxytoca*	2	2	ND	ND	ND	2
	*Enterobacter cloacae*	2	2	1	ND	1	1
*armA*	*Escherichia coli*	5	2	2	5	ND	ND
	*Klebsiella pneumonia*	10	10	9	5	3	
**TMP-SMX resistance genes**							
*sul1*	*Escherichia coli*	35	31	29	10	8	1
	*Klebsiella pneumonia*	46	41	38	30	13	5
	*Proteus mirabilis*	6	6	3	1	2	4
	*Citrobacter freundii*	7	5	4	2	1	ND
	*Klebsiella oxytoca*	6	5	3	4	2	1
	*Enterobacter cloacae*	5	4	3	2	2	2
	*Proteus vulgaris*	2	2	ND	ND	1	ND
	*Morganella morganii*	1	1	ND	ND	ND	ND
sul2	*Escherichia coli*	9	7	4	4	ND	ND
	*Klebsiella pneumonia*	6	6	3	2	2	2
	*Klebsiella oxytoca*	1	1	ND	ND	ND	ND
	*Enterobacter cloacae*	1	1	1	ND	ND	1
*dfrA1*	*Escherichia coli*	20	18	20	5	5	1
	*Klebsiella pneumonia*	28	26	23	18	10	2
	*Proteus mirabilis*	2	2	1	1	1	2
	*Citrobacter freundii*	4	2	3	2	ND	ND
	*Klebsiella oxytoca*	1	1	ND	ND	ND	ND
	*Enterobacter cloacae*	2	2	1	ND	1	1
	*Proteus vulgaris*	1	1	ND	ND	1	ND
	*Morganella morganii*	1	1	ND	ND	ND	ND

ND: Not detected.

**Table 5:** Comparison of origin of strain and type of resistance genes.

		Origin of Strain		
Type of Resistance Gene		Wound Specimens	Respiratory Tract Specimens	Blood and Body Fluids
		N = 97 (%)	N = 44 (%)	N = 30 (%)
ESBL				
	*TEM*	85 (87.62)	35 (79.54)	25 (83.33)
	*CTX-M*	70 (72.16)	34 (77.27)	22 (73.33)
	*SHV*	40 (41.23)	19 (43.18)	16 (53.33)
	*PER*	29 (29.89)	ND	4 (13.3)
	*VEB*	14 (14.43)	1 (2.27)	2 (6.6)
PMQR				
	*qnrA*	13 (13.4)	ND	ND
	*qnrB*	19 (19.58)	17 (38.63)	24 (80)
	*qnrC*	ND	ND	4 (13.3)
	*qnrD*	27 (27.83)	ND	ND
	*qnrS*	26 (26.8)	ND	ND
	*aac(6′)-lb-cr*	76 (78.35)	8 (18.18)	24 (80)
	*oqxA*	43 (44.33)	16 (36.36)	28 (93.33)
	*oqxB*	39 (40.2)	27 (61.36)	28 (93.33)
	*qepA*	ND	ND	5 (16.66)
AMEs				
	*aac(3)-Ib*	11 (11.34)	ND	ND
	*aac(3)-Ia*	6 (6.19)	ND	1 (3.33)
	*aac(3)-IIa*	44 (45.36)	ND	3 (10)
	*aac(6′)-Ib*	62 (63.91)	3 (6.82)	20 (66.67)
	*ant(2”)-Ia*	9 (9.28)	ND	ND
	*ant(3”)-Ia*	33 (34)	ND	13 (41.94)
	*ant(4”)-IIa*	13 (13.4)	ND	ND
	*aph(3′)-Ia*	39 (40.21)	ND	9 (30)
	*aph(3”)-Ia*	1 (1.03)	ND	ND
	*aph(3”)-Ib*	25 (25.77)	ND	15 (50)
	*armA*	8 (8.24)	4 (9.09)	3 (10)
TMP-SMX resistance gene				
	*sul 1*	78 (80.41)	3 (6.82)	27 (90)
	*sul 2*	8 (8.24)	ND	9 (30)
	*dfr A*	59 (60.82)	ND	ND

ND: Not detected.

## Supplementary Materials

The following are available online at https://www.mdpi.com/2079-6382/9/12/884/s1, Table S1: Antibiotic resistance pattern of *Enterobacterales* isolates tested, Table S2: Distribution of *Enterobacterales* isolates in various specimens studied, Table S3: Distribution of plasmid mediated quinolone resistance (PMQR) genes in extended spectrum beta-lactamase producing *Enterobacterales* isolates, Table S4: Distribution of aminoglycoside modifying enzyme (AME) genes in extended spectrum beta-lactamase producing *Enterobacterales*, Table S5: Distribution of TMP-SMX resistant genes in extended spectrum beta-lactamase producing *Enterobacterales*, Table S6: Distribution plasmid mediated quinolone resistance genes in extended spectrum beta-lactamase producing *Enterobacterales* isolates and its comparison with minimum inhibitory concentrations of fluoroquinolones tested, Table S7: Distribution of aminoglycoside modifying genes in extended spectrum beta-lactamase producing *Enterobacterales* isolates and its comparison with minimum inhibitory concentrations of aminoglycosides tested, Table S8: Distribution of trimethoprim-sulfamethoxazole (TMP-SMX) resistance genes in extended spectrum beta-lactamase producing *Enterobacterales* isolates and its comparison with minimum inhibitory concentrations of TMP-SMX tested.
