# Supplementary material for: Monitoring of Non-β-Lactam Antibiotic Resistance-Associated Genes in ESBL Producing Enterobacterales Isolates
Source: Antibiotics (Basel). 2020 Dec 9;9(12):884. doi: 10.3390/antibiotics9120884 (PMC7764327; doi:10.3390/antibiotics9120884)
Supplement: Supplementary file 1 [file antibiotics-09-00884-s001.pdf]

antibiotics MDPI

Supplementary Materials

Monitoring of Non-β-Lactam Antibiotic Resistance-Associated Genes in ESBL Producing *Enterobacterales* Isolates

Pottathil Shinu [1,*], Rajesh Bareja [2], Anroop B. Nair [3], Vashishth Mishra [2], Snawar Hussain [1], Katharigatta N. Venugopala [3,4], Nagaraja Sreeharsha [3,5], Mahesh Attimarad [3] and Sahibzada Tasleem Rasool [1]

[1] Department of Biomedical Sciences, College of Clinical Pharmacy, King Faisal University, Al-Ahsa 31982, Saudi Arabia; shhussain@kfu.edu.sa (S.H.); srasool@kfu.edu.sa (S.T.R.)
[2] Department of Microbiology, Faculty of Medicine, Government Medical College and Hospital, Badaun 243601, India; rajeshbareja@gmail.com (R.B.); vashishtmmishra38@gmail.com (V.M.)
[3] Department of Pharmaceutical Sciences, College of Clinical Pharmacy, King Faisal University, Al-Ahsa 31982, Saudi Arabia; anair@kfu.edu.sa (A.B.N.); kvenugopala@kfu.edu.sa (K.N.V.); sharsha@kfu.edu.sa (N.S.); mattimarad@kfu.edu.sa (M.A.)
[4] Department of Biotechnology and Food Technology, Durban University of Technology, Durban 4001, South Africa
[5] Department of Pharmaceutics, Vidya Siri College of Pharmacy, Off Sarjapura Road, Bangaluru 560035, India
* Correspondence: spottathail@kfu.edu.sa (P.S.); Tel.: +966-559-732794

Table S1. Antibiotic resistance pattern of *Enterobacterales* isolates tested.

**Organism (N=186)**	**AMP (N=73) %**	**CFZ (N=186) %**	**AMC (N=161) %**	**CTX (N=186) %**	**FEP (N=186)**	**CAZ (N=186)**	**CRO (N=186)**	**FOX (N=164)**	**CPD (N=180)**	**CXM (N=186)**	**ZOX (N=186)**	**IPM (N=186)**	**MEM (N=186)**	**ATM (N=186)**	**GEN (N=186)**	**TOB (N=186)**	**AMK (N=186)**	**K (N=186)**	**CIP (N=186)**	**LVX (N=186)**	**NAL (N=186)**	**GAT (N=186)**	**MXF (N=186)**	**TMP-SXT (N=186)**
Escherichia coli (N=58)	55(75.34)	49(26.34)	38(23.6)	48(31.17)	46(24.73)	52(27.96)	50(26.88)	41(25)	52(28.89)	50(26.88)	47(25.26)	22(11.83)	27(14.52)	54(29.03)	40(21.5)	45(24.19)	32(17.2)	40(21.5)	46(24.73)	40(21.5)	46(24.73)	47(33.57)	42(22.58)	56(30.1)
Klebsiella pneumonia (N=74)	-	69 (37.01)	64(83.1)	63(33.87)	41(22.04)	66(35.48)	71(43.29)	64(39.02)	66(36.67)	69(37.09)	67(36)	30(16.13)	32(17.2)	68(36.56)	46(24.73)	50(26.88)	39(20.96)	36(19.35)	65(34.94)	56(301)	64(34.4)	62(33.33)	59(31.72)	67(36.02)

Proteus mirabilis (N=15)	15(20.55)	15(8.06)	12(7.45)	9(10.87)	10(5.38)	13(6.99)	8(4.3)	11(6.7)	7(3.89)	9(4.84)	10(5.38)	12(6.45)	11(5.91)	12(6.45)	7(3.76)	5(2.68)	3(1.61)	8(4.3)	8(4.3)	11(5.91)	11(5.91)	12(6.45)	12(6.45)	12(6.45)
Citrobacter freundii (N=13)	-	12(6.45)	-	13(6.98)	11(5.91)	11(5.91)	13(6.98)	-	10(5.56)	11(5.91)	10(5.37)	7(3.76)	7(3.76)	10(5.38)	5(2.68)	7(3.76)	3(1.61)	6(3.2)	8(4.3)	7(3.76)	5(2.68)	6(3.22)	7(3.76)	11(5.91)
Klebsiella oxytoca (N=11)	-	10(5.38)	8(4.9)	8(4.3)	9(4.84)	11(5.91)	11(5.91)	7(4.27)	11(6.11)	10(5.37)	8(4.3)	6(3.23)	6(3.22)	11(5.91)	4(2.15)	6(3.22)	5(2.69)	6(3.2)	6(3.22)	8(4.3)	8(4.3)	7(3.76)	7(3.76)	9(4.83)
Enterobacter cloacae (N=9)	-	9(4.84)	-	9(4.84)	5(2.69)	9(4.83)	7(3.76)	-	9(5)	7(3.76)	9(4.84)	3(1.61)	3(1.61)	9(4.84)	8(4.3)	7(3.76)	6(3.23)	9(4.84)	4(2.15)	4(2.15)	4(2.15)	3(1.61)	3(1.61)	9(4.83)
Proteus vulgaris(N =3)	-	3(1.62)	2(1.24)	2(1.07)	1(0.53)	2(1.07)	2(1.07)	2(1.22)	-	2(1.07)	2(1.07)	-	1(0.54)	2(1.07)	1(0.53)	2(1.07)	-	1(0.53)	1(0.53)	2(1.07)	3(1.61)	1(0.53)	1(0.53)	2(1.07)
Morganella morganii (N=3)	-	3(1.62	-	2(1.07)	1(0.53)	3(1.61)	2(1.07)	1(0.6)	-	2(1.07)	2(1.07)	-	-	1(0.54)	2(1.07)	2(1.07)	2(1.07)	2(1.07)	2(0.01)	2(1.07)	2(1.07)	2(1.07)	2(1.07)	3(1.61)
Total	70(95.89)	170(91.4)	124(77.01)	154(82.8)	124(66.67)	167(89.78)	164(88.17)	126(76.83)	155(86.11)	160(86.02)	(155)83.3	80(43.01)	87(46.77)	167(89.78)	113(60.75)	124(66.67)	90(48..39)	(108)58.06	140(75.27)	130(69.89)	143(76.88)	145(75.27)	133(71.51)	169(90.86)

AMP- Ampicillin, CFZ- cefazolin, AMC- Amoxicillin- clavulanic acid, CTX –Cefotaxime, FEP- Cefepime, CAZ- Ceftazidime, CRO- Ceftriaxone, FOX- Cefoxitin, CPD- Cefpodoxime, CXM –Cefuroxime, ZOX- Ceftazidime, IPM- Imipenem, MEM- Meropenem, ATM- Aztreonam, GEN- Gentamicin, TOB- Tobramycin, AMK- Amikacin, K- Kanamycin, CIP- Ciprofloxacin, LVX- Levofloxacin, NAL- Nalidixic acid, GAT- Gatifloxacin, MXF- Moxifloxacin, TMP-SXT- Trimethoprim-sulfamethoxazole

Table S2. Distribution of *Enterobacterales* isolates in various specimens studied.

Sr. No.	Year of isolation	Key for Isolates	Ward/Unit/Department	specimen
1	2018	EC1	Orthopaedic	Wound
2	2018	EC2	Surgery	Wound
3	2018	EC3	ENT	Wound
4	2018	EC4	Orthopaedic	Wound
5	2018	EC5	Orthopaedic	Wound
6	2018	EC6	Paediatric	Pus
7	2018	EC7	RICU	pus
8	2018	EC8	Surgery	Wound
9	2018	EC9	ENT	Wound
10	2018	EC10	PICU	Central line
11	2018	EC11	Surgery	Ascitic fluid
12	2018	EC12	Burn ICU	Wound
13	2018	EC13	Orthopaedic	surgical drain
14	2018	EC14	Orthopaedic	Wound
15	2018	EC15	Orthopaedic	Wound
16	2018	EC16	Paediatrics	Wound
17	2018	EC17	Emergency	Wound
18	2018	EC18	Orthopaedic	Pus
19	2018	EC19	Surgery	Wound
20	2018	EC20	PICU	Wound
21	2018	EC21	Burn ICU	Wound
22	2018	EC22	Orthopaedic	Wound
23	2019	EC23	Orthopaedic	Wound
24	2019	EC24	Surgery	Blood
25	2019	EC25	orthopaedic	Blood
26	2019	EC26	Orthopaedic	Blood
27	2019	EC27	Surgery	Blood
28	2019	EC28	Paediatric	Blood
29	2019	EC29	Orthopaedic	Blood
30	2019	EC30	Surgery	Peritoneal fluid
31	2019	EC31	Emergency	Blood
32	2019	EC32	Surgery	Blood
33	2019	EC33	Orthopaedic	Blood
34	2019	EC34	Orthopaedic	Blood
35	2019	EC35	Paediatrics	Blood
36	2019	EC36	Surgery	Blood
37	2019	EC37	Orthopaedic	Nasal Swab
38	2019	EC38	Burn ICU	Sputum
39	2019	EC39	Chest and TB	Endotracheal aspirate
40	2019	EC40	ENT	Nasal Swab
41	2019	EC41	Chest and TB	Sputum
42	2019	EC42	Burn ICU	Endotracheal aspirate
43	2019	EC43	Chest and TB	Sputum
44	2019	EC44	Chest and TB	Sputum

45	2019	EC45	General Medicine	Endotracheal aspirate
46	2019	EC46	Chest and TB	Sputum
47	2019	EC47	Chest and TB	Sputum
48	2019	EC48	orthopaedic	Sputum
49	2019	EC49	Burn ICU	Endotracheal aspirate
50	2019	EC50	Chest and TB	Sputum
51	2019	EC51	Chest and TB	Sputum
52	2019	EC52	paediatric	Endotracheal aspirate
53	2019	EC53	Chest and TB	Sputum
54	2019	EC54	Emergency	Endotracheal aspirate
55	2019	EC55	ENT	Sputum
56	2019	EC56	Surgery	Endotracheal aspirate
57	2019	EC57	Chest and TB	Sputum
58	2019	EC58	orthopaedics	Endotracheal aspirate
59	2018	KLP1	Burn ICU	Wound
60	2018	KLP2	Emergency	Wound
61	2018	KLP3	Surgery	Wound
62	2018	KLP4	orthopaedics	Pus
63	2018	KLP5	Burn ICU	Wound
64	2018	KLP6	Burn ICU	Wound
65	2018	KLP7	orthopaedics	Wound
66	2018	KLP8	Burn ICU	Wound
67	2018	KLP9	Surgery	Wound
68	2018	KLP10	Surgery	Wound
69	2018	KLP11	General Medicine	Wound
70	2018	KLP12	Burn ICU	Wound
71	2018	KLP13	Orthopaedic	Pus
72	2018	KLP14	Surgery	Wound
73	2018	KLP15	Emergency	Wound
74	2018	KLP16	Orthopaedic	Wound
75	2018	KLP17	Surgery	Wound
76	2018	KLP18	ENT	Wound
77	2018	KLP19	Paediatric	Wound
78	2018	KLP20	Orthopaedic	surgical drainage
79	2018	KLP21	Burn ICU	Wound
80	2018	KLP22	Emergency	Wound
81	2018	KLP23	orthopaedic	Wound
82	2018	KLP24	PICU	Pus
83	2018	KLP25	Surgery	Pus
84	2018	KLP26	Burn ICU	Wound
85	2018	KLP27	ENT	Wound
86	2018	KLP28	Surgery	Wound
87	2018	KLP29	Emergency	Blood
88	2018	KLP30	Surgery	Blood
89	2018	KLP31	Surgery	Blood
90	2018	KLP32	orthopaedics	Blood

91	2018	KLP33	paediatrics	Blood
92	2018	KLP34	Orthopaedic	Blood
93	2018	KLP35	Burn ICU	Blood
94	2018	KLP36	NICU	CSF
95	2018	KLP37	orthopaedic	Blood
96	2018	KLP38	Burn ICU	Blood
97	2018	KLP39	Emergency	Blood
98	2018	KLP40	orthopaedic	Blood
99	2018	KLP41	PICU	Blood
100	2018	KLP42	Surgery	Blood
101	2018	KLP43	NICU	CSF
102	2018	KLP44	ENT	Nasal Swab
103	2018	KLP45	orthopaedics	Sputum
104	2018	KLP46	ENT	Endotracheal aspirate
105	2018	KLP47	Chest and TB	Sputum
106	2019	KLP48	Chest and TB	Sputum
107	2019	KLP49	paediatrics	Endotracheal aspirate
108	2019	KLP50	Chest and TB	Endotracheal aspirate
109	2019	KLP51	Chest and TB	Sputum
110	2019	KLP52	Chest and TB	Sputum
111	2019	KLP53	ENT	Nasal Swab
112	2019	KLP54	Chest and TB	Sputum
113	2019	KLP55	Chest and TB	Endotracheal aspirate
114	2019	KLP56	Chest and TB	Sputum
115	2019	KLP57	Emergency	Sputum
116	2019	KLP58	Chest and TB	Endotracheal aspirate
117	2019	KLP59	Chest and TB	Sputum
118	2019	KLP60	Chest and TB	Sputum
119	2019	KLP61	paediatrics	Sputum
120	2019	KLP62	Chest and TB	Endotracheal aspirate
121	2019	KLP63	General Medicine	Sputum
122	2019	KLP64	Chest and TB	Endotracheal aspirate
123	2019	KLP65	Chest and TB	Sputum
124	2019	KLP66	Emergency	Sputum
125	2019	KLP67	Chest and TB	Sputum
126	2019	KLP68	Emergency	Sputum
127	2019	KLP69	Chest and TB	Sputum
128	2019	KLP70	paediatrics	Sputum
129	2019	KLP71	ENT	Endotracheal aspirate
130	2019	KLP72	paediatrics	Sputum
131	2019	KLP73	Chest and TB	Endotracheal aspirate
132	2019	KLP74	Chest and TB	Sputum
133	2018	PLM1	ENT	Wound
134	2018	PLM2	paediatrics	Wound
135	2018	PLM3	Emergency	Wound
136	2018	PLM4	ENT	Wound

137	2018	PLM5	Surgery	Urinary catheter
138	2018	PLM6	Emergency	Wound
139	2019	PLM7	ENT	Wound
140	2019	PLM8	orthopaedics	Wound
141	2019	PLM9	ENT	Wound
142	2019	PLM10	Surgery	Wound
143	2019	PLM11	ENT	Wound
144	2019	PLM12	paediatrics	Ascitic fluid
145	2019	PLM13	orthopaedics	Wound
146	2019	PLM14	Emergency	Wound
147	2019	PLM15	Surgery	Wound
148	2018	CRF1	Burn ICU	Wound
149	2018	CRF2	orthopaedics	surgical drainage
150	2018	CRF3	Burn ICU	Wound
151	2018	CRF4	Emergency	Wound
152	2018	CRF5	Surgery	Wound
153	2018	CRF6	General Medicine	Wound
154	2019	CRF7	Burn ICU	Wound
155	2019	CRF8	Burn ICU	Wound
156	2019	CRF9	orthopaedics	Wound
157	2019	CRF10	Chest and TB	Endo tracheal aspirate
158	2019	CRF11	Burn ICU	Blood
159	2019	CRF12	orthopaedics	Blood
160	2019	CRF13	Surgery	Blood
161	2018	KLO1	ENT	Nasal Swab
162	2018	KLO2	paediatrics	Wound
163	2018	KLO3	Burn ICU	Wound
164	2018	KLO4	Burn ICU	Wound
165	2018	KLO5	orthopaedics	Wound
166	2018	KLO6	orthopaedics	surgical drainage
167	2019	KLO7	paediatrics	Wound
168	2019	KLO8	ENT	Wound
169	2019	KLO9	General Medicine	Wound
170	2019	KLO10	paediatrics	Sputum
171	2019	KLO11	Chest and TB	Endotracheal aspirate
172	2018	ERC1	Emergency	Wound
173	2018	ERC2	ENT	Wound
174	2018	ERC3	Surgery	Wound
175	2018	ERC4	Emergency	Wound
176	2019	ERC5	orthopaedics	Wound
177	2019	ERC6	PICU	Pus
178	2019	ERC7	Surgery	Wound
179	2019	ERC8	Burn ICU	Wound
180	2019	ERC9	Chest and TB	Sputum
181	2018	PSV1	PICU	Pus
182	2018	PSV2	Emergency Medicine	Wound

183	2019	PSV3	orthopaedics	Wound
184	2019	MAM1	Burn ICU	Wound
185	2019	MAM2	Emergency	Wound
186	2019	MAM3	Chest and TB	Sputum

EC- *Escherichia coli*, KLP- *Klebsiella pneumonia*, PLM- *Proteus mirabilis*, CRF- *Citrobacter freundii*, KLO - *Klebsiella oxytoca*, ERC – *Enterobacter cloacae*, PSV- *Proteus vulgaris*, MAM- *Morganella morganii*

Table S3. Distribution of plasmid mediated quinolone resistance (PMQR) genes in extended spectrum beta-lactamase producing *Enterobacterales* isolates.

**Isolate Key**	**Year of isolation**	**Cephalosporin susceptibility**					**Fluoroquinolone susceptibility**					**ESBL encoding genes**					**PMQR encoding genes**								
		CTX	CAZ	CRO	CPD	ATM	CIP	LVX	NAL	GAT	MXF	*TEM*	*CTXM*	*SHV*	*PER*	*VEB*	*qnrA*	*qnrB*	*qnrC*	*qnrD*	*qnrS*	*aac (6')-lb-cr*	*oqxA*	*oqxB*	*qepA*
EC12	2018	R	R	R	R	R	R	R	R	R	R	+	+	+	+	-	-	-	-	+	-	+	-	-	-
EC13	2018	R	R	R	R	R	R	R	R	R	R	+	+	+	+	-	-	-	-	+	-	+	-	-	-
EC11	2018	R	R	R	R	R	R	R	R	R	R	+	+	+	-	-	-	-	-	+	+	+	-	-	-
EC6	2018	R	R	R	R	R	R	R	R	R	R	+	+	-	+	-	-	-	-	-	+	+	-	-	-
EC7	2018	R	R	R	R	R	R	R	R	R	R	+	+	-	+	-	-	-	-	-	+	+	-	-	-
EC4	2018	R	R	R	R	R	R	R	R	R	R	+	+	-	-	+	-	-	-	-	+	+	-	-	-
EC14	2018	R	R	R	R	R	R	R	R	R	R	+	+	+	-	-	-	-	-	+	-	+	-	-	-
EC21	2018	R	R	R	R	R	R	R	R	R	R	+	+	+	+	-	-	-	-	-	-	+	-	-	-
EC22	2018	R	R	R	R	R	R	R	R	R	R	+	+	+	+	-	-	-	-	-	-	+	-	-	-
EC23	2019	R	R	R	R	R	R	R	R	R	R	+	+	+	+	-	-	-	-	-	-	+	-	-	-
EC33	2019	R	R	R	R	R	R	R	R	R	R	+	+	+	-	-	-	+	-	-	-	-	+	+	+
EC41	2019	R	R	R	R	R	R	ND	R	R	R	+	+	+	-	-	-	-	-	-	-	-	-	+	-
EC42	2019	R	R	R	R	R	R	ND	R	R	R	+	+	+	-	-	-	-	-	-	-	-	-	+	-
EC43	2019	R	R	R	R	R	R	ND	R	R	ND	+	+	+	-	-	-	-	-	-	-	-	-	+	-
EC44	2019	R	R	R	R	R	R	ND	R	R	ND	+	+	+	-	-	-	-	-	-	-	-	-	+	-
EC20	2018	R	R	R	R	R	R	R	R	R	R	+	+	+	-	-	-	-	-	-	-	+	-	-	-
EC8	2018	R	R	R	R	R	R	R	R	R	R	+	+	-	-	-	-	-	-	-	+	+	-	-	-
EC9	2018	R	R	R	R	R	R	R	R	R	R	+	+	-	-	-	-	-	-	-	+	+	-	-	-
EC10	2018	R	R	R	R	R	R	R	R	R	R	+	+	-	-	-	-	-	-	-	+	+	-	-	-
EC3	2018	R	R	R	R	R	R	R	R	R	R	+	+	-	-	-	+	-	-	-	+	+	-	-	-
EC5	2018	R	R	R	R	R	R	R	R	R	R	+	+	-	-	-	-	-	-	-	+	+	-	-	-
EC15	2018	R	R	R	R	R	R	R	R	R	R	+	+	-	-	-	-	-	-	+	-	+	-	-	-
EC16	2018	R	R	R	R	R	R	R	R	R	R	+	+	-	-	-	-	-	-	+	-	+	-	-	-
EC17	2018	R	R	R	R	R	R	R	R	R	R	+	+	-	-	-	-	-	-	+	-	+	-	-	-
EC18	2018	R	R	R	R	R	R	R	R	R	R	+	+	-	-	-	-	-	-	-	-	+	-	-	-

EC19	2018	R	R	R	R	R	R	R	R	R	R	+	+	-	-	-	-	-	-	-	-	+	-	-	-
EC24	2019	R	R	R	R	R	R	R	R	R	R	+	+	-	-	-	-	-	-	-	-	+	+	+	-
EC25	2019	R	R	R	R	R	R	R	R	R	R	+	+	-	-	-	-	-	-	-	-	+	+	+	-
EC26	2019	R	R	R	R	R	R	R	R	R	R	+	+	-	-	-	-	-	-	-	-	+	+	+	-
EC27	2019	R	R	R	R	R	R	R	R	R	R	+	+	-	-	-	-	-	-	-	-	+	+	+	-
EC28	2019	R	R	R	R	R	R	R	R	R	R	+	+	-	-	-	-	+	+	-	-	+	+	+	-
EC29	2019	R	R	R	R	R	R	R	R	R	R	+	+	-	-	-	-	+	+	-	-	+	+	+	-
EC36	2019	R	R	R	R	R	R	R	R	R	R	-	+	+	-	-	-	+	-	-	-	-	+	+	-
EC37	2019	R	R	R	R	R	R	R	R	R	R	-	+	+	-	-	-	+	-	-	-	-	+	+	-
EC39	2019	R	R	R	R	R	R	R	R	R	R	+	-	+	-	-	-	+	-	-	-	-	-	+	-
EC40	2019	R	R	R	R	R	R	R	R	R	R	+	-	+	-	-	-	-	-	-	-	-	-	+	-
EC1	2018	R	R	R	R	R	R	R	R	R	R	-	+	-	+	-	+	-	-	-	+	+	-	-	-
EC32	2019	R	R	R	R	R	R	R	R	R	R	+	-	+	-	-	+	+	-	-	-	+	+	+	+
EC30	2019	R	R	R	R	R	R	R	R	R	R	+	-	-	-	-	+	+	+	-	-	+	+	+	-
EC31	2019	R	R	R	R	R	R	R	R	R	R	+	-	-	-	-	+	+	+	-	-	+	+	+	+
EC34	2019	R	R	R	R	R	R	R	R	R	R	-	-	+	-	-	+	+	-	-	-	-	+	+	+
EC35	2019	R	R	R	R	R	R	R	R	R	R	-	-	+	-	-	+	+	-	-	-	-	+	+	+
EC38	2019	R	R	R	R	R	R	R	R	R	R	-	-	+	-	-	+	+	-	-	-	-	-	+	-
EC2	2018	R	R	R	R	R	R	R	R	R	R	-	+	-	-	-	+	-	-	-	+	+	-	-	-
KLP1	2018	R	R	R	R	R	R	R	R	R	R	+	-	+	+	+	-	-	-	+	+	+	+	+	-
KLP8	2018	R	R	R	R	R	R	R	R	R	R	+	+	+	+	-	-	-	-	+	-	+	+	+	-
KLP12	2018	R	R	R	R	R	R	R	R	R	R	+	+	+	+	-	-	-	-	+	-	+	+	+	-
KLP16	2018	R	R	R	R	R	R	R	R	R	R	+	+	+	+	-	-	-	-	+	-	+	+	+	-
KLP17	2018	R	R	R	R	R	R	R	R	R	R	+	+	+	+	-	-	-	-	+	-	+	+	+	-
KLP4	2018	R	R	R	R	R	R	R	R	R	R	+	+	-	+	-	-	-	-	+	-	+	+	+	-
KLP6	2018	R	R	R	R	R	R	R	R	R	R	+	+	-	-	+	-	-	-	+	-	+	+	+	-
KLP7	2018	R	R	R	R	R	R	R	R	R	R	+	+	+	-	-	-	-	-	+	-	+	+	+	-
KLP9	2018	R	R	R	R	R	R	R	R	R	R	+	+	+	-	-	-	-	-	+	-	+	+	+	-
KLP11	2018	R	R	R	R	R	R	R	R	R	R	+	-	+	+	-	-	-	-	+	-	+	+	+	-

KLP15	2018	R	R	R	R	R	R	R	R	R	R	+	+	+	-	-	-	-	-	+	-	+	+	+	-
KLP18	2018	R	R	R	R	R	R	R	R	R	R	+	+	+	-	-	-	-	-	-	-	+	+	+	-
KLP19	2018	R	R	R	R	R	R	R	R	R	R	+	+	+	-	-	-	-	-	-	-	+	+	+	-
KLP20	2018	R	R	R	R	R	R	R	R	R	R	+	+	+	-	-	-	-	-	-	-	+	+	+	-
KLP26	2018	R	R	R	R	R	R	R	R	R	R	+	+	+	-	-	-	+	-	-	-	+	+	+	-
KLP27	2018	R	R	R	R	R	R	R	R	R	R	+	+	+	-	-	-	+	-	-	-	+	+	+	-
KLP28	2018	R	R	R	R	R	R	R	R	R	R	+	+	+	-	-	-	+	-	-	-	+	+	+	-
KLP29	2018	R	R	R	R	R	R	R	R	R	R	+	+	+	-	-	-	+	-	-	-	+	+	+	-
KLP30	2018	R	R	R	R	R	R	R	R	R	R	+	+	+	-	-	-	+	-	-	-	+	+	+	-
KLP41	2018	R	R	R	R	R	R	R	R	R	R	+	+	+	-	-	-	+	-	-	-	+	+	+	-
KLP43	2018	R	R	R	R	R	R	R	R	R	R	+	+	+	-	-	-	+	-	-	-	+	+	+	-
KLP44	2018	R	R	R	R	R	R	R	R	R	R	+	+	+	-	-	-	+	-	-	-	+	+	+	-
KLP45	2018	R	R	R	R	R	R	R	R	R	R	+	+	+	-	-	-	+	-	-	-	+	+	+	-
KLP36	2018	R	R	R	R	R	R	R	R	R	R	+	+	+	-	-	-	+	-	-	-	+	+	+	-
KLP32	2018	R	R	R	R	R	R	R	R	R	R	+	-	+	+	-	-	+	-	-	-	+	+	+	-
KLP33	2018	R	R	R	R	R	R	R	R	R	R	+	-	+	+	-	-	+	-	-	-	+	+	+	-
KLP34	2018	R	R	R	R	R	R	R	R	R	R	+	-	+	+	-	-	+	-	-	-	+	+	+	-
KLP22	2018	R	R	R	R	R	R	R	R	R	R	+	+	-	+	-	-	+	-	-	-	+	+	+	-
KLP23	2018	R	R	R	R	R	R	R	R	R	R	+	+	-	+	-	-	+	-	-	-	+	+	+	-
KLP21	2018	R	R	R	R	R	R	R	R	R	R	+	-	-	+	-	-	-	-	-	-	+	+	+	-
KLP42	2018	R	R	R	R	R	R	R	R	R	R	-	+	+	-	+	-	+	-	-	-	+	+	+	-
KLP37	2018	R	R	R	R	R	R	R	R	R	R	+	+	-	-	+	-	+	-	-	-	+	+	+	-
KLP5	2018	R	R	R	R	R	R	R	R	R	R	+	+	-	-	-	-	-	-	+	-	+	+	+	-
KLP10	2018	R	R	R	R	R	R	R	R	R	R	+	+	-	-	-	-	-	-	+	-	+	+	+	-
KLP24	2018	R	R	R	R	R	R	R	R	R	R	+	+	-	-	-	-	+	-	-	-	+	+	+	-
KLP25	2018	R	R	R	R	R	R	R	R	R	R	+	+	-	-	-	-	+	-	-	-	+	+	+	-
KLP31	2018	R	R	R	R	R	R	R	R	R	R	+	+	-	-	-	-	+	-	-	-	+	+	+	-
KLP38	2018	R	R	R	R	R	R	R	R	R	R	+	+	-	-	-	-	+	-	-	-	+	+	+	-
KLP39	2018	R	R	R	R	R	R	R	R	R	R	+	+	-	-	-	-	+	-	-	-	+	+	+	-

KLP40	2018	R	R	R	R	R	R	R	R	R	R	+	+	-	-	-	-	+	-	-	-	+	+	+	-
KLP47	2018	R	R	R	R	R	R	R	R	R	R	+	+	-	-	-	-	+	-	-	-	+	+	+	-
KLP48	2019	R	R	R	R	R	R	R	R	R	R	+	+	-	-	-	-	+	-	-	-	+	+	+	-
KLP49	2019	R	R	R	R	R	R	R	R	R	R	+	+	-	-	-	-	+	-	-	-	+	+	+	-
KLP50	2019	R	R	R	R	R	R	R	R	R	R	+	+	-	-	-	-	+	-	-	-	+	+	+	-
KLP51	2019	R	R	R	R	R	R	R	R	R	R	+	+	-	-	-	-	+	-	-	-	+	+	+	-
KLP55	2019	R	R	R	R	R	R	R	R	R	R	+	+	-	-	-	-	+	-	-	-	-	+	+	-
KLP56	2019	R	R	R	R	R	R	R	R	R	R	+	+	-	-	-	-	+	-	-	-	-	+	+	-
KLP57	2019	R	R	R	R	R	R	ND	R	R	R	+	+	-	-	-	-	+	-	-	-	-	+	+	-
KLP59	2019	R	R	R	R	R	R	ND	R	R	R	+	+	-	-	-	-	-	-	-	-	-	-	+	-
KLP60	2019	R	R	R	R	R	R	ND	R	R	ND	+	+	-	-	-	-	-	-	-	-	-	-	+	-
KLP61	2019	R	R	R	R	R	R	ND	R	R	ND	+	+	-	-	-	-	-	-	-	-	-	-	+	-
KLP62	2019	R	R	R	R	R	R	ND	R	R	ND	+	+	-	-	-	-	-	-	-	-	-	-	+	-
KLP13	2018	R	R	R	R	R	R	R	R	R	R	-	+	+	-	-	-	-	-	+	-	+	+	+	-
KLP14	2018	R	R	R	R	R	R	R	R	R	R	-	+	+	-	-	-	-	-	+	-	+	+	+	-
KLP35	2018	R	R	R	R	R	R	R	R	R	R	-	+	+	-	-	-	+	-	-	-	+	+	+	-
KLP2	2018	R	R	R	R	R	R	R	R	R	R	+		+	-	-	-	-	-	+	+	+	+	+	-
KLP52	2019	R	R	R	R	R	R	R	R	R	R	-	+	-	-	-	-	+	-	-	-	-	+	+	-
KLP53	2019	R	R	R	R	R	R	R	R	R	R	-	+	-	-	-	-	+	-	-	-	-	+	+	-
KLP54	2019	R	R	R	R	R	R	R	R	R	R	-	+	-	-	-	-	+	-	-	-	-	+	+	-
KLP3	2018	R	R	R	R	R	R	R	R	R	R	+		-	-	-	-	-	-	+	+	+	+	+	-
KLP58	2019	R	R	R	R	R	R	ND	R	R	R	-	+	-	-	-	-	-	-	-	-	-	+	+	-
KLP46	2018	R	R	R	R	R	R	R	R	R	R	-	+	-	-	+	-	+	-	-	-	+	+	+	-
PLM2	2018	R	R	R	R	R	R	R	R	R	R	+	+	+	-	+	-	-	-	-	+	+	-	+	-
PLM6	2018	R	R	R	R	R	R	R	R	R	R	+		-	+	+	+	-	-	-	+	+	+	+	-
PLM8	2019	R	R	R	S	R	R	R	R	R	R	+	+	+	-	-	+	-	-	-	+	-	+	+	-
PLM10	2019	R	R	S	S	R	I	R	R	R	R	+	+	-	+	-	+	-	-	-	-	-	+	-	-
PLM11	2019	R	R	S	S	R	I	R	R	R	R	+	+	-	-	-	+	-	-	-	-	-	+	-	-
PLM12	2019	R	R	S	S	R	I	ND	ND	R	R	-	+	-	-	+	+	-	-	-	-	-	+	-	-

PLM1	2018	R	R	R	R	R	R	R	R	R	R	+	-	-	+	+	-	-	-	-	+	+	-	+	-
PLM4	2018	R	R	R	R	R	R	R	R	R	R	+	+	-	-	+	-	-	-	-	+	+	+	+	-
PLM3	2018	R	R	R	R	R	R	R	R	R	R	+	+	-	-	-	-	-	-	-	+	+	-	+	-
PLM5	2018	R	R	R	R	R	R	R	R	R	R	+	-	-	-	-	+	-	-	-	+	+	+	+	-
PLM7	2019	R	R	R	R	R	R	R	R	R	R	+	-	-	-	+	+	-	-	-	+	+	+	+	-
PLM9	2019	R	R	S	S	R	S	R	R	R	R	+	-	-	-	+	+	-	-	-	+	-	+	-	-
CRF3	2018	R	R	R	R	R	R	R	R	R	R	+	+	-	-	-	-	+	-	-	-	+	-	-	-
CRF6	2018	R	R	R	R	R	R	R	ND	R	R	+	+	-	-	-	-	+	-	-	-	+	-	-	-
CRF1	2018	R	R	R	R	R	R	R	R	R	R	-	+	+	-	-	-	-	-	-	-	+	-	-	-
CRF2	2018	R	R	R	R	R	R	R	R	R	R	-	+	+	-	-	-	+	-	-	-	+	-	-	-
CRF4	2018	R	R	R	R	R	R	R	R	R	R	+	-	-	-	-	-	+	-	-	-	+	-	-	-
CRF5	2018	R	R	R	R	R	R	R	R	R	R	+	-	-	+	-	-	+	-	-	-	+	-	-	-
CRF7	2019	R	R	R	R	R	R	R	ND		R	+	-	-	-	-	-	+	-	-	-	+	-	-	-
KLO6	2018	R	R	R	R	R	R	R	R	R	R	+	+	+	+	-	-	-	-	-	-	+	-	-	-
KLO7	2019	R	R	R	R	R	S	R	R	R	R	+	+	+	-	-	-	-	-	-	-	+	-	-	-
KLO5	2018	R	R	R	R	R	R	R	R	R	R	+	+	+	-	-	-	+	-	-	-	+	-	-	-
KLO4	2018	R	R	R	R	R	R	R	R	R	R	-	+	+	+	-	-	+	-	-	-	+	+	-	-
KLO3	2018	R	R	R	R	R	R	R	R	R	R	+	-	+	-	-	-	+	-	+	-	+	+	+	-
KLO2	2018	R	R	R	R	R	R	R	R	R	R	+	-	-	-	+	-	+	-	+	-	+	+	+	-
KLO1	2018	R	R	R	R	R	R	R	R	R	R	+	-	-	-	-	-	+	-	+	-	-	+	+	-
ERC1	2018	R	R	R	R	R	R	R	R	R	R	+	+	-	-	+	-	-	-	-	+	+	+	-	-
ERC2	2018	R	R	R	R	R	R	R	R	R	R	+	-	-	+	-	-	-	-	-	+	+	+	-	-
ERC3	2018	R	R	R	R	R	R	R	R	R	R	+	-	-	-	-	-	-	-	-	+	-	-	-	-
PSV1	2018	R	R	S	-	R	R	R	ND	R	R	+	-	-	+	-	+	-	-	-	-	+	-	-	-
PSV2	2018	R	R	I	-	R	S	R	R	ND	ND	+	-	-	-	-	+	-	-	-	-	+	-	-	-
PSV3	2019	S	S	R	-	S	S	ND	R	ND	ND	+	-	-	-	-	-	-	-	-	-	+	-	-	-
MAM1	2019	R	R	R	-	R	R	R	R	R	R	+	-	-	-	-	-	+	-	-	-	-	-	-	-

Abbreviations used in Tables; EC- *Escherichia coli*, KLP- *Klebsiella pneumonia*, PLM- *Proteus mirabilis* ,CRF- *Citrobacter freundii*, KLO - *Klebsiella oxytoca*, ERC – *Enterobacter cloacae*, PSV- *Proteus vulgaris*, MAM- *Morganella morganii*, R- Resistance, S- Susceptible, I –Intermediate Resistance, ND- Not done

Table S4. Distribution of aminoglycoside modifying enzyme (AME) genes in extended spectrum beta-lactamase producing *Enterobacterales*.

Key	Year	B-lactam susceptibility					Aminoglycoside susceptibility				ESBL					AME									
		CTX	CAZ	CRO	CPD	ATM	GEN	TOB	AMK	K	*bla* $_{TEM}$	*bla* $_{CTX-M}$	*bla* $_{SHV}$	*bla* $_{PER}$	*bla* $_{VEB}$	*aac(3)-Ib*	*aac(3)-Ia*	*aac(3)-IIa*	*aac(6')-Ib*	*ant(2")-Ia*	*ant(3")-Ia*	*ant(4")-IIa*	*aph(3')-Ia*	*aph(3")-Ib*	*armA*
EC12	2018	R	R	R	R	R	R	R	R	R	+	+	+	+	-	-	-	+	+	-	-	-	+	-	-
EC13	2018	R	R	R	R	R	R	R	R	R	+	+	+	+	-	-	-	+	+	-	-	+	+	-	-
EC21	2018	R	R	R	R	R	R	R	R	R	+	+	+	+	-	-	+	-	+	-	+	-	+	+	-
EC22	2018	R	R	R	R	R	R	R	R	R	+	+	+	+	-	-	+	-	+	-	+	-	+	+	-
EC23	2019	R	R	R	R	R	R	R	R	R	+	+	+	+	-	-	+	-	+	-	+	-	+	+	-
EC20	2018	R	R	R	R	R	R	R	R	R	+	+	+	-	-	-	+	-	+	-	+	+	+	+	-
EC14	2018	R	R	R	R	R	R	R	R	R	+	+	+	-	-	-	-	+	+	-	-	+	+	-	-
EC11	2018	R	R	R	R	R	R	R	R	R	+	+	+	-	-	-	-	+	+	-	-	-	+	-	-
EC6	2018	R	R	R	R	R	R	R	R	R	+	+	-	+	-	-	-	+	+	-	-	-	-	-	-
EC7	2018	R	R	R	R	R	R	R	R	R	+	+	-	+	-	-	-	+	+	-	-	-	-	-	-
EC4	2018	R	R	R	R	R	R	R	R	R	+	+	-	-	+	-	-	+	+	-	-	-	-	-	-
EC33	2019	R	R	R	R	R	R	R	S	R	+	+	+	-	-	-	-			-	+	-	-	+	-
EC3	2018	R	R	R	R	R	R	R	R	R	+	+	-	-	-	-	-	+	+	-	-	-	-	-	-
EC5	2018	R	R	R	R	R	R	R	R	R	+	+	-	-	-	-	-	+	+	-	-	-	-	-	-
EC8	2018	R	R	R	R	R	R	R	R	R	+	+	-	-	-	-	-	+	+	-	-	-	-	-	-
EC9	2018	R	R	R	R	R	R	R	R	R	+	+	-	-	-	-	-	+	+	-	-	-	-	-	-
EC10	2018	R	R	R	R	R	R	R	R	R	+	+	-	-	-	-	-	+	+	-	-	-	+	-	-
EC15	2018	R	R	R	R	R	R	R	R	R	+	+	-	-	-	-	-	+	+	-	+	+	+	-	-
EC16	2018	R	R	R	R	R	R	R	R	R	+	+	-	-	-	-	-	+	+	-	+	+	+	-	-
EC17	2018	R	R	R	R	R	R	R	R	R	+	+	-	-	-	-	-	+	+	-	+	+	+	+	-

EC18	2018	R	R	R	R	R	R	R	R	R	+	+	-	-	-	+	-	+	+	-	+	+	+	+	-
EC19	2018	R	R	R	R	R	R	R	R	R	+	+	-	-	-	+	+	-	+	-	+	+	+	+	-
EC24	2019	R	R	R	R	R	R	R	R	R	+	+	-	-	-	-	+	-	+	-	+	-	+	+	-
EC25	2019	R	R	R	R	R	R	R	R	R	+	+	-	-	-	-	-	-	+	-	+	-	+	+	-
EC26	2019	R	R	R	R	R	R	R	R	R	+	+	-	-	-	-	-	-	+	-	+	-	-	+	-
EC27	2019	R	R	R	R	R	R	R	R	R	+	+	-	-	-	-	-	-	+	-	+	-	-	+	-
EC28	2019	R	R	R	R	R	R	R	R	R	+	+	-	-	-	-	-	-	+	-	+	-	-	+	-
EC29	2019	R	R	R	R	R	R	R	R	R	+	+	-	-	-	-	-	-	-	-	+	-	-	+	-
EC32	2019	R	R	R	R	R	R	R	R	R	+	-	+	-	-	-	-	-	-	-	+	-	-	+	-
EC39	2019	R	R	R	R	R	R	R	S	R	+	-	+	-	-	-	-	-	-	-	-	-	-	-	+
EC40	2019	R	R	R	R	R	R	R	S	R	+	-	+	-	-	-	-	-	-	-	-	-	-	-	+
EC36	2019	R	R	R	R	R	R	R	S	R	-	+	+	-	-	-	-	-	-	-	+	-	-	+	+
EC37	2019	R	R	R	R	R	R	R	S	R	-	+	+	-	-	-	-	-	-	-	-	-	-	-	+
EC1	2018	R	R	R	R	R	R	R	R	R	-	+	-	+	-	-	-	+	+	-	-	-	-	-	-
EC2	2018	R	R	R	R	R	R	R	R	R	-	+	-	-	-	-	-	+	+	-	-	-	-	-	-
EC30	2019	R	R	R	R	R	R	R	R	R	+	-	-	-	-	-	-	-	-	-	+	-	-	+	-
EC31	2019	R	R	R	R	R	R	R	R	R	+	-	-	-	-	-	-	-	-	-	+	-	-	+	-
EC34	2019	R	R	R	R	R	R	R	S	R	-	-	+	-	-	-	-	-	-	-	+	-	-	+	-
EC35	2019	R	R	R	R	R	R	R	S	R	-	-	+	-	-	-	-	-	-	-	+	-	-	+	-
EC38	2019	R	R	R	R	R	R	R	S	R	-	-	+	-	-	-	-	-	-	-	-	-	-	-	+
KLP1	2018	R	R	R	R	R	R	R	R	R	+	-	+	+	+	+	-	-	+	-	-	-	-	-	-
KLP16	2018	R	R	R	R	R	R	R	R	R	+	+	+	+	-	-	-	+	+	+	+	-	+	+	-
KLP17	2018	R	R	R	R	R	R	R	R	R	+	+	+	+	-	-	-	+	+	+	+	-	+	+	-
KLP8	2018	R	R	R	R	R	R	R	R	R	+	+	+	+	-	-	-	-	+	-	+	-	+	-	-

KLP 12	2018	R	R	R	R	R	R	R	R	R	+	+	+	+	-	-	-	-	+	-	+	-	+	-	-
KLP 4	2018	R	R	R	R	R	R	R	R	R	+	+	-	+	-	+	-	-	+	-	-	-	-	-	-
KLP 18	2018	R	R	R	R	R	R	R	R	R	+	+	+	-	-	-	-	+	+	+	+	-	+	+	-
KLP 7	2018	R	R	R	R	R	R	R	R	R	+	+	+	-	-	-	-	-	+	-	+	-	+	-	-
KLP 9	2018	R	R	R	R	R	R	R	R	R	+	+	+	-	-	-	-	-	+	-	+	-	+	-	-
KLP 11	2018	R	R	R	R	R	R	R	R	R	+	-	+	+	-	-	-	-	+	-	+	-	+	-	-
KLP 15	2018	R	R	R	R	R	R	R	R	R	+	+	+	-	-	-	-	+	+	+	+	-	+	-	-
KLP 19	2018	R	R	R	R	R	R	R	R	R	+	+	+	-	-	-	-	+	+	-	+	-	+	+	-
KLP 20	2018	R	R	R	R	R	R	R	R	R	+	+	+	-	-	-	-	+	+	-	+	-	+	+	-
KLP 22	2018	R	R	R	R	R	R	R	R	R	+	+	-	+	-	-	-	+	+	-	+	-	+	+	+
KLP 23	2018	R	R	R	R	R	R	R	R	R	+	+	-	+	-	-	-	+	+	-	+	-	+	+	+
KLP 26	2018	R	R	R	R	R	R	R	R	R	+	+	+	-	-	-	-	+	+	-	-	+	+	+	+
KLP 27	2018	R	R	R	R	R	R	R	R	R	+	+	+	-	-	-	-	+	+	-	-	+	+	+	+
KLP 28	2018	R	R	R	R	R	R	R	R	R	+	+	+	-	-	-	-	+	+	-	-	-	+	+	+
KLP 29	2018	R	R	R	R	R	R	R	R	R	+	+	+	-	-	-	-	+	+	-	-	-	+	+	+
KLP 30	2018	R	R	R	R	R	R	R	R	R	+	+	+	-	-	-	-	+	+	-	-	-	+	+	+
KLP 32	2018	R	R	R	R	R	R	R	R	R	+	-	+	+	-	-	-	-	+	-	-	-	+	-	-
KLP 33	2018	R	R	R	R	R	R	R	R	R	+	-	+	+	-	-	-	-	+	-	-	-	+	-	-
KLP 34	2018	R	R	R	R	R	R	R	R	R	+	-	+	+	-	-	-	-	+	-	-	-	+	-	-
KLP 41	2018	R	R	R	R	R	R	R	S	S	+	+	+	-	-	-	-	-	+	-	-	-	-	-	-
KLP 42	2018	R	R	R	R	R	R	R	S	S	-	+	+	-	+	-	-	-	+	-	-	-	-	-	-
KLP 43	2018	R	R	R	R	R	R	R	S	S	+	+	+	-	-	-	-	-	+	-	-	-	-	-	-
KLP 44	2018	R	R	R	R	R	R	R	S	S	+	+	+	-	-	-	-	-	+	-	-	-	-	-	-
KLP 45	2018	R	R	R	R	R	R	R	S	S	+	+	+	-	-	-	-	-	+	-	-	-	-	-	-

KLP 36	2018	R	R	R	R	R	R	R	R	R	+	+	+	-	-	-	-	-	+	-	-	-	-	-	-
KLP 6	2018	R	R	R	R	R	R	R	R	R	+	+	-	-	+	-	-	-	+	-	-	-	+	-	-
KLP 37	2018	R	R	R	R	R	R	R	R	S	+	+	-	-	+	-	-	-	+	-	-	-	-	-	-
KLP 2	2018	R	R	R	R	R	R	R	R	R	+	-	+	-	-	+	-	-	+	-	-	-	-	-	-
KLP 5	2018	R	R	R	R	R	R	R	R	R	+	+	-	-	-	-	-	-	+	-	-	-	-	-	-
KLP 24	2018	R	R	R	R	R	R	R	R	R	+	+	-	-	-	-	-	+	+	-	-	+	+	+	+
KLP 25	2018	R	R	R	R	R	R	R	R	R	+	+	-	-	-	-	-	+	+	-	-	+	+	+	+
KLP 10	2018	R	R	R	R	R	R	R	R	R	+	+	-	-	-	-	-	-	+	-	+	-	+	-	-
KLP 13	2018	R	R	R	R	R	R	R	R	R	-	+	+	-	-	-	-	+	+	+	+	-	+	-	-
KLP 14	2018	R	R	R	R	R	R	R	R	R	-	+	+	-	-	-	-	+	+	+	+	-	+	-	-
KLP 3	2018	R	R	R	R	R	R	R	R	R	+	-	-	-	-	+	-	-	+	-	-	-	-	-	-
KLP 21	2018	R	R	R	R	R	R	R	R	R	+	-	-	+	-	-	-	+	+	-	+	-	+	+	+
KLP 31	2018	R	R	R	R	R	R	R	R	R	+	+	-	-	-	-	-	+	+	-	-	-	+	-	-
KLP 35	2018	R	R	R	R	R	R	R	R	R	-	+	+	-	-	-	-	-	+	-	-	-	+	-	-
KLP 38	2018	R	R	R	R	R	R	R	R	S	+	+	-	-	-	-	-	-	+	-	-	-	-	-	-
KLP 39	2018	R	R	R	R	R	R	R	R	S	+	+	-	-	-	-	-	-	+	-	-	-	-	-	-
KLP 40	2018	R	R	R	R	R	R	R	S	S	+	+	-	-	-	-	-	-	+	-	-	-	-	-	-
KLP 46	2018	R	R	R	R	R	R	R	S	S	-	+	-	-	+	-	-	-	+	-	-	-	-	-	-
PL M2	2018	R	R	R	R	R	R	R	R	R	+	+	+	-	+	-	-	-	+	-	+	-	-	-	-
PL M1	2018	R	R	R	R	R	R	R	R	R	+	-	-	+	+	-	-	+	+	-	+	+	+	+	-
PL M3	2018	R	R	R	R	R	R	R	R	R	+	+	-	-	-	-	-	-	+	-	-	-	-	-	-
CRF 1	2018	R	R	R	R	R	R	R	R	R	-	+	+	-	-	+	+	-	+	-	-	-	-	-	-
CRF 2	2018	R	R	R	R	R	R	R	R	R	-	+	+	-	-	+	-	-	+	-	-	-	-	-	-
CRF 3	2018	R	R	R	R	R	R	R	R	R	+	+	-	-	-	+	-	-	-	-	-	-	-	-	-

CRF4	2018	R	R	R	R	R	R	R	S	R	+	-	-	-	-	-	-	+	-	-	+	-	-	-	-
CRF5	2018	R	R	R	R	R	R	R	S	R	+	-	-	+	-	-	-	+	-	-	+	-	-	-	-
CRF6	2018	R	R	R	R	R	S	R	S	R	+	+	-	-	-	-	-	+	-	-	+	-	-	-	-
KLO4	2018	R	R	R	R	R	R	R	R	R	-	+	+	+	-	-	-	-	+	-	-	-	-	-	-
KLO2	2018	R	R	R	R	R	R	R	R	R	+	-	-	-	+	-	-	+	+	-	-	-	-	+	-
KLO3	2018	R	R	R	R	R	R	R	R	R	+	-	+	-	-	-	-	-	+	-	-	-	-	-	-
KLO1	2018	R	R	R	R	R	R	R	R	R	+	-	-	-	-	+	-	+	+	+	-	-	+	+	-
ERC1	2018	R	R	R	R	R	R	R	R	R	+	+	-	-	+	+	-	+	+	+	+	-	-	+	-
ERC2	2018	R	R	R	R	R	R	R	R	R	+	-	-	+	-	-	-	+	-	+	-	-	-	+	-
ERC3	2018	R	R	R	R	R	R	R	R	R	+	-	-	-	-	-	-	+	-	-	-	-	-	-	-
PSV1	2018	R	R	S	R	R	R	S	S	R	+	-	-	+	-	-	-	-	-	-	+	-	-	-	-
MAM1	2019	R	R	R	R	R	R	R	R	R	+	-	-	-	-	-	-	+	+	-	-	-	-	-	-

Abbreviations used in Tables ; EC- *Escherichia coli*, KLP- *Klebsiella pneumonia*, PLM- *Proteus mirabilis* ,CRF- *Citrobacter freundii*, KLO - *Klebsiella oxytoca*, ERC – *Enterobacter cloacae*, PSV- *Proteus vulgaris*, MAM- *Morganella morganii*, R- Resistance, S- Susceptible, I –Intermediate Resistance, ND- Not done

Table S5. Distribution of TMP-SMX resistant genes in extended spectrum beta-lactamase producing *Enterobacterales*.

Key	Year	B-lactam susceptibility					TMP-SMX susceptibility	ESBL encoding genes detected					TMP-SMX		
		CTX	CAZ	CRO	CPD	ATM	TMP-SMX	*bla* $_{TEM}$	*bla* $_{CTX-M}$	*bla* $_{SHV}$	*bla* $_{PER}$	*bla* $_{VEB}$	*sul 1*	*sul 2*	*dfr A*
EC12	2018	R	R	R	R	R	R	+	+	+	+	-	+	-	+
EC13	2018	R	R	R	R	R	R	+	+	+	+	-	+	-	+
EC21	2018	R	R	R	R	R	R	+	+	+	+	-	+	-	-
EC22	2018	R	R	R	R	R	R	+	+	+	+	-	+	-	-
EC20	2018	R	R	R	R	R	R	+	+	+	-	-	+	-	+
EC14	2018	R	R	R	R	R	R	+	+	+	-	-	+	-	+
EC11	2018	R	R	R	R	R	R	+	+	+	-	-	+	-	+
EC6	2018	R	R	R	R	R	R	+	+	-	+	-	+	-	+
EC7	2018	R	R	R	R	R	R	+	+	-	+	-	+	-	+
EC4	2018	R	R	R	R	R	R	+	+	-	-	+	+	-	+
EC23	2019	R	R	R	R	R	R	+	+	+	+	-	+	-	-
EC33	2019	R	R	R	R	R	R	+	+	+	-	-	+	+	-
EC27	2019	R	R	R	R	R	R	+	+	-	-	-	+	+	-
EC28	2019	R	R	R	R	R	R	+	+	-	-	-	+	+	-
EC29	2019	R	R	R	R	R	R	+	+	-	-	-	+	+	-
EC15	2018	R	R	R	R	R	R	+	+	-	-	-	+	-	+
EC16	2018	R	R	R	R	R	R	+	+	-	-	-	+	-	+
EC17	2018	R	R	R	R	R	R	+	+	-	-	-	+	-	+
EC18	2018	R	R	R	R	R	R	+	+	-	-	-	+	-	+
EC19	2018	R	R	R	R	R	R	+	+	-	-	-	+	-	+
EC8	2018	R	R	R	R	R	R	+	+	-	-	-	+	-	+
EC9	2018	R	R	R	R	R	R	+	+	-	-	-	+	-	+
EC10	2018	R	R	R	R	R	R	+	+	-	-	-	+	-	+
EC5	2018	R	R	R	R	R	R	+	+	-	-	-	+	-	+
EC3	2018	R	R	R	R	R	R	+	+	-	-	-	+	-	+
EC24	2019	R	R	R	R	R	R	+	+	-	-	-	+	-	-

EC25	2019	R	R	R	R	R	R	+	+	-	-	-	+	-	-
EC26	2019	R	R	R	R	R	R	+	+	-	-	-	+	-	-
EC30	2019	R	R	R	R	R	R	+	-	-	-	-	+	+	-
EC31	2019	R	R	R	R	R	R	+	-	-	-	-	+	+	-
EC32	2019	R	R	R	R	R	R	+	-	+	-	-	+	+	-
EC34	2019	R	R	R	R	R	R	-	-	+	-	-	+	+	-
EC35	2019	R	R	R	R	R	R	-	-	+	-	-	+	+	-
EC1	2018	R	R	R	R	R	R	-	+	-	+	-	+	-	+
EC2	2018	R	R	R	R	R	R	-	+	-	-	-	+	-	+
KLP8	2018	R	R	R	R	R	R	+	+	+	+	-	+	-	+
KLP12	2018	R	R	R	R	R	R	+	+	+	+	-	+	-	+
KLP1	2018	R	R	R	R	R	R	+	-	+	+	+	+	+	+
KLP16	2018	R	R	R	R	R	R	+	+	+	+	-	+	-	+
KLP17	2018	R	R	R	R	R	R	+	+	+	+	-	+	-	+
KLP7	2018	R	R	R	R	R	R	+	+	+	-	-	+	-	+
KLP11	2018	R	R	R	R	R	R	+	-	+	+	-	+	-	+
KLP15	2018	R	R	R	R	R	R	+	+	+	-	-	+	-	+
KLP18	2018	R	R	R	R	R	R	+	+	+	-	-	+	-	+
KLP19	2018	R	R	R	R	R	R	+	+	+	-	-	+	-	+
KLP20	2018	R	R	R	R	R	R	+	+	+	-	-	+	-	+
KLP22	2018	R	R	R	R	R	R	+	+	-	+	-	+	-	+
KLP23	2018	R	R	R	R	R	R	+	+	-	+	-	+	-	+
KLP26	2018	R	R	R	R	R	R	+	+	+	-	-	+	-	+
KLP27	2018	R	R	R	R	R	R	+	+	+	-	-	+	-	+
KLP28	2018	R	R	R	R	R	R	+	+	+	-	-	+	-	+
KLP29	2018	R	R	R	R	R	R	+	+	+	-	-	+	-	-
KLP30	2018	R	R	R	R	R	R	+	+	+	-	-	+	-	-
KLP32	2018	R	R	R	R	R	R	+	-	+	+	-	+	-	-
KLP33	2018	R	R	R	R	R	R	+	-	+	+	-	+	-	-
KLP34	2018	R	R	R	R	R	R	+	-	+	+	-	+	-	-

KLP36	2018	R	R	R	R	R	R	+	+	+	-	-	+	-	-
KLP43	2018	R	R	R	R	R	R	+	+	+	-	-	+	-	-
KLP44	2018	R	R	R	R	R	R	+	+	+	-	-	+	-	-
KLP45	2018	R	R	R	R	R	R	+	+	+	-	-	+	-	-
KLP42	2018	R	R	R	R	R	R	-	+	+	-	+	+	-	-
KLP41	2018	R	R	R	R	R	R	+	+	+	-	-	+	-	-
KLP4	2018	R	R	R	R	R	R	+	+	-	+	-	+	+	+
KLP9	2018	R	R	R	R	R	R	+	+	+	-	-	+	-	+
KLP5	2018	R	R	R	R	R	R	+	+	-	-	-	+	+	+
KLP6	2018	R	R	R	R	R	R	+	+	-	-	+	+	+	+
KLP13	2018	R	R	R	R	R	R	-	+	+	-	-	+	-	+
KLP14	2018	R	R	R	R	R	R	-	+	+	-		+	-	+
KLP37	2018	R	R	R	R	R	R	+	+	-	-	+	+	-	-
KLP38	2018	R	R	R	R	R	R	+	+	-	-	-	+	-	-
KLP39	2018	R	R	R	R	R	R	+	+	-	-	-	+	-	-
KLP40	2018	R	R	R	R	R	R	+	+	-	-	-	+	-	-
KLP2	2018	R	R	R	R	R	R	+	-	+	-	-	+	+	+
KLP10	2018	R	R	R	R	R	R	+	+	-	-	-	+	-	+
KLP21	2018	R	R	R	R	R	R	+	-	-	+	-	+	-	+
KLP24	2018	R	R	R	R	R	R	+	+	-	-	-	+	-	+
KLP25	2018	R	R	R	R	R	R	+	+	-	-	-	+	-	+
KLP31	2018	R	R	R	R	R	R	+	+	-	-	-	+	-	-
KLP35	2018	R	R	R	R	R	R	-	+	+	-	-	+	-	-
KLP3	2018	R	R	R	R	R	R	+	-	-	-	-	+	+	+
KLP46	2018	R	R	R	R	R	R	-	+	-	-	+	+	-	-
PLM2	2018	R	R	R	R	R	R	+	+	+	-	+	+	-	+
PLM4	2018	R	R	R	R	R	R	+	+	-	-	+	+	-	-
PLM6	2018	R	R	R	R	R	R	+	-	-	+	+	+	-	-
PLM1	2018	R	R	R	R	R	R	+	-	-	+	+	+	-	+
PLM3	2018	R	R	R	R	R	R	+	+	-	-	-	+	-	-

PLM5	2018	R	R	R	R	R	R	+	-	-	-	-	+	-	_
CRF1	2018	R	R	R	R	R	R	-	+	+	-	-	+	-	+
CRF2	2018	R	R	R	R	R	R	-	+	+	-	-	+	-	+
CRF3	2018	R	R	R	R	R	R	+	+	-	-	-	+	-	+
CRF5	2018	R	R	R	R	R	R	+	-	-	+	-	+	-	-
CRF6	2018	R	R	R	R	R	R	+	+	-	-	-	+	-	-
CRF4	2018	R	R	R	R	R	R	+	-	-	-	-	+	-	+
CRF7	2019	R	R	R	R	R	R	+	-	-	-	-	+	-	-
KLO6	2018	R	R	R	R	R	R	+	+	+	+	-	+	-	-
KLO4	2018	R	R	R	R	R	R	-	+	+	+	-	+	-	-
KLO5	2018	R	R	R	R	R	R	+	+	+	-	-	+	-	-
KLO3	2018	R	R	R	R	R	R	+	-	+	-	-	+	-	-
KLO1	2018	R	R	R	R	R	R	+	-	-	-	-	+	+	+
KLO2	2018	R	R	R	R	R	R	+	-	-	-	+	+	-	-
ERC5	2019	R	R	R	R	R	R	+	+	+	+	+	+	-	-
ERC1	2018	R	R	R	R	R	R	+	+	-	-	+	+	+	+
ERC2	2018	R	R	R	R	R	R	+	-	-	+	-	+	-	+
ERC3	2018	R	R	R	R	R	R	+	-	-	-	-	+	-	-
ERC4	2018	R	R	R	R	R	R	-	+	+	-	-	+	-	-
PSV1	2018	R	R	S	-	R	R	+	-	-	+	-	+	-	+
PSV2	2018	R	R	S	-	R	R	+	-	-	-	-	+	-	-
MAM1	2019	R	R	R	-	R	R	+	-	-	-	-	+	-	-

Abbreviations used in Tables ; EC- *Escherichia coli*, KLP- *Klebsiella pneumonia*, PLM- *Proteus mirabilis* ,CRF- *Citrobacter freundii*, KLO - *Klebsiella oxytoca*, ERC – *Enterobacter cloacae*, PSV- *Proteus vulgaris*, MAM- *Morganella morganii* , R- Resistance , S- Susceptible ,I –Intermediate Resistance , ND- Not done

Table S6. Distribution plasmid mediated quinolone resistance genes in extended spectrum beta-lactamase producing *Enterobacterales* isolates and its comparison with minimum inhibitory concentrations of fluoroquinolones tested.

**Key For Isolates tested**	**Year of isolation**	**MICs of Fluoroquinolones**					**PMQR encoding genes**								
		CIP	LVX	NAL	GAT	MXF	*qnrA*	*qnrB*	*qnrC*	*qnrD*	*qnrS*	*aac (6')-lb-cr*	*oqxA*	*oqxB*	*qepA*
EC7	2018	1024	1024	64	512	256	-	-	-	-	+	+	-	-	-
EC22	2018	1024	1024	256	256	512	-	-	-	-	+	+	-	-	-
EC42	2019	1024	512	256	1024	512	-	-	-	+	-	+	-	-	-
EC5	2018	1024	1024	64	256	256	-	-	-	-	-	+	-	-	-
EC18	2018	1024	1024	256	512	512	-	-	-	-	-	+	+	+	-
EC26	2019	1024	512	256	256	512	-	+	+	-	-	+	+	+	-
EC40	2019	1024	1024	64	512	1024	-	+	-	-	-	-	+	+	-
EC31	2019	1024	1024	256	512	256	-	-	-	-	-	-	-	+	-
EC35	2019	1024	1024	64	512	512	-	-	-	-	-	-	-	+	-
EC6	2018	512	64	64	128	64	-	-	-	-	+	+	-	-	-
EC4	2018	512	256	64	256	128	-	-	-	-	+	+	-	-	-
EC14	2018	256	512	128	128	64	-	-	-	-	+	+	-	-	-
EC21	2018	512	256	64	256	128	-	-	-	-	+	+	-	-	-
EC23	2019	512	256	128	128	256	-	-	-	-	+	+	-	-	-
EC33	2019	256	512	64	512	512	-	-	-	+	+	+	-	-	-
EC41	2019	256	512	64	256	512	-	-	-	+	-	+	-	-	-
EC15	2018	512	256	64	256	512	-	-	-	-	-	+	-	-	-
EC16	2018	256	512	128	64	256	-	-	-	-	-	+	-	-	-
EC17	2018	512	256	64	128	512	-	-	-	-	-	+	+	+	-
EC19	2018	512	256	128	512	512	-	-	-	-	-	+	+	+	-
EC24	2019	256	512	64	64	128	-	-	-	-	-	+	+	+	-
EC25	2019	256	512	64	32	64	-	+	+	-	-	+	+	+	-
EC32	2019	256	512	128	256	512	-	+	-	-	-	-	-	+	-
EC30	2019	512	256	64	128	256	-	+	-	-	-	-	-	+	-
EC1	2018	512	256	64	128	512	-	+	-	-	-	-	+	+	-
EC38	2019	512	256	64	256	256	-	-	-	-	-	-	-	+	-

EC2	2018	256	512	128	1024	512	-	-	-	-	-	-	-	+	-
EC20	2018	128	256	64	512	128	-	-	-	+	-	+	-	-	-
EC28	2019	128	256	64	128	256	-	+	+	-	-	+	+	+	+
EC12	2018	128	32	32	64	16	+	-	-	-	+	+	-	-	-
EC13	2018	128	32	32	64	32	+	-	-	-	+	+	-	-	-
EC43	2019	128	64	128	128	256	-	-	-	+	-	+	-	-	-
EC44	2019	128	128	64	128	128	-	-	-	+	-	+	-	-	-
EC11	2018	256	128	128	256	64	+	-	-	-	+	+	-	-	-
EC27	2019	128	64	128	128	64	-	+	+	-	-	+	+	+	-
EC3	2018	32	64	32	32	64	-	-	-	-	-	+	-	-	-
EC8	2018	32	32	16	16	64	-	-	-	+	-	+	-	-	-
EC9	2018	32	16	32	32	16	-	-	-	-	-	+	-	-	-
EC10	2018	32	32	64	32	64	-	-	-	-	-	+	-	-	-
EC37	2019	32	32	64	64	32	-	+	-	-	-	-	+	+	+
EC39	2019	32	64	32	64	128	-	+	-	-	-	-	+	+	+
EC34	2019	32	64	32	128	32	-	-	-	-	-	-	-	+	-
EC29	2019	32	32	16	32	64	-	+	-	-	-	+	+	+	+
EC36	2019	32	16	32	32	16	-	+	-	-	-	-	+	+	+
EC45	2019	8	ND	16	ND	ND	-	-	-	-	-	-	-	-	-
EC46	2019	4	ND	16	ND	ND	-	-	-	-	-	-	-	-	-
KLP12	2018	1024	1024	256	512	256	-	-	-	+	+	+	+	+	-
KLP6	2018	1024	512	256	1024	256	-	-	-	+	-	+	+	+	-
KLP26	2018	1024	1024	64	1024	1024	-	-	-	+	-	+	+	+	-
KLP29	2018	1024	512	256	512	512	-	-	-	-	-	+	+	+	-
KLP23	2018	1024	1024	64	512	1024	-	+	-	-	-	+	+	+	-
KLP39	2018	1024	1024	64	1024	512	-	+	-	-	-	+	+	+	-
KLP48	2019	1024	512	256	1024	512	-	+	-	-	-	+	+	+	-
KLP52	2019	1024	512	256	512	512	-	+	-	-	-	-	+	+	-
KLP42	2018	256	512	128	512	1024	-	+	-	-	-	+	+	+	-
KLP35	2018	512	256	64	512	256	-	+	-	-	-	-	+	+	-

KLP40	2018	512	256	64	256	256	-	+	-	-	-	+	+	+	-
KLP21	2018	512	256	64	512	512	-	+	-	-	-	+	+	+	-
KLP27	2018	512	256	64	256	256	-	-	-	+	-	+	+	+	-
KLP28	2018	256	512	128	1024	512	-	-	-	+	-	+	+	+	-
KLP16	2018	512	256	128	256	512	-	-	-	+	-	+	+	+	-
KLP17	2018	256	512	64	256	512	-	-	-	+	-	+	+	+	-
KLP4	2018	256	512	64	512	1024	-	-	-	+	-	+	+	+	-
KLP1	2018	256	512	128	1024	512	-	-	-	+	+	+	+	+	-
KLP8	2018	512	256	64	256	512	-	-	-	+	+	+	+	+	-
KLP47	2018	256	512	128	512	512	-	+	-	-	-	+	+	+	-
KLP2	2018	256	512	128	256	256	-	+	-	-	-	-	+	+	-
KLP49	2019	128	64	128	64	64	-	+	-	-	-	+	+	+	-
KLP50	2019	128	128	64	256	128	-	+	-	-	-	+	+	+	-
KLP55	2019	128	64	128	64	64	-	+	-	-	-	+	+	+	-
KLP56	2019	128	128	64	128	256	-	+	-	-	-	+	+	+	-
KLP60	2019	128	128	64	256	128	-	+	-	-	-	+	+	+	-
KLP46	2018	128	128	64	128	64	-	-	-	-	-	-	-	+	-
KLP7	2018	128	64	128	64	64	-	-	-	+	-	+	+	+	-
KLP9	2018	128	128	64	128	128	-	-	-	+	-	+	+	+	-
KLP11	2018	128	256	64	256	256	-	-	-	+	-	+	+	+	-
KLP45	2018	128	128	64	256	128	-	+	-	-	-	+	+	+	-
KLP36	2018	128	256	64	256	512	-	+	-	-	-	+	+	+	-
KLP53	2019	128	64	128	64	128	-	-	-	-	-	-	+	+	-
KLP54	2019	128	128	64	128	128	-	-	-	-	-	-	-	+	-
KLP3	2018	128	256	64	256	512	-	-	-	-	-	-	-	+	-
KLP30	2018	128	64	128	128	64	-	-	-	-	-	+	+	+	-
KLP41	2018	128	128	64	128	128	-	-	-	-	-	+	+	+	-
KLP43	2018	128	256	64	256	512	-	-	-	-	-	+	+	+	-
KLP51	2019	128	256	64	512	256	-	+	-	-	-	+	+	+	-
KLP57	2019	128	256	64	256	256	-	+	-	-	-	+	+	+	-

KLP61	2019	128	256	64	128	512	-	+	-	-	-	+	+	+	-
KLP37	2018	128	64	128	64	128	-	+	-	-	-	+	+	+	-
KLP5	2018	128	128	64	128	256	-	+	-	-	-	+	+	+	-
KLP10	2018	128	256	64	512	256	-	+	-	-	-	+	+	+	-
KLP15	2018	32	32	16	128	64	-	-	-	+	-	+	+	+	-
KLP18	2018	32	16	32	16	32	-	-	-	+	-	+	+	+	-
KLP19	2018	32	32	64	32	32	-	-	-	+	-	+	+	+	-
KLP20	2018	32	64	32	64	128	-	-	-	+	-	+	+	+	-
KLP24	2018	32	32	16	32	64	-	+	-	-	-	+	+	+	-
KLP25	2018	32	16	32	16	32	-	+	-	-	-	+	+	+	-
KLP31	2018	32	32	64	32	32	-	+	-	-	-	+	+	+	-
KLP38	2018	32	64	32	64	128	-	+	-	-	-	+	+	+	-
KLP32	2018	32	32	16	32	32	-	+	-	-	-	+	+	+	-
KLP33	2018	32	16	32	16	16	-	+	-	-	-	+	+	+	-
KLP34	2018	32	32	64	32	32	-	+	-	-	-	+	+	+	-
KLP22	2018	32	64	32	64	64	-	+	-	-	-	+	+	+	-
KLP44	2018	32	32	16	64	32	-	+	-	-	-	+	+	+	-
KLP59	2019	32	32	16	32	64	-	+	-	-	-	+	+	+	-
KLP62	2019	32	32	16	32	32	-	+	-	-	-	-	+	+	-
KLP13	2018	32	16	32	32	16	-	+	-	-	-	-	+	+	-
KLP14	2018	32	32	64	32	64	-	+	-	-	-	-	+	+	-
KLP58	2019	32	32	16	64	128	-	-	-	-	-	-	-	+	-
KLP63	2019	8	ND	32	128	32	-	-	-	-	-	-	-	-	-
KLP64	2019	8	ND	16	64	16	-	-	-	-	-	-	-	-	-
KLP65	2019	4	ND	ND	16	64	-	-	-	-	-	-	-	-	-
PLM10	2019	1024	1024	64	256	512	-	-	-	-	+	+	+	+	-
PLM11	2019	512	256	64	256	512	+	-	-	-	+	+	+	+	-
PLM12	2019	256	512	128	512	256	+	-	-	-	+	+	+	+	-
PLM1	2018	128	64	128	64	128	+	-	-	-	+	+	+	+	-
PLM4	2018	128	128	64	128	256	+	-	-	-	+	-	+	+	-

PLM3	2018	128	256	64	512	256	+	-	-	-	+	-	+	-	-
PLM2	2018	32	16	32	64	32	-	-	-	-	+	+	-	+	-
PLM6	2018	32	32	64	32	16	-	-	-	-	+	+	-	+	-
PLM8	2019	32	64	32	128	128	-	-	-	-	+	+	-	+	-
PLM5	2018	32	32	16	32	32	+	-	-	-	-	-	+	-	-
PLM7	2019	32	32	64	32	64	+	-	-	-	-	-	+	-	-
PLM9	2019	32	64	32	64	64	+	-	-	-	-	-	+	-	-
CRF1	2018	128	256	64	256	512	-	+	-	-	-	+	-	-	-
CRF3	2018	128	64	128	64	128	-	-	-	-	-	+	-	-	-
CRF6	2018	128	128	64	256	128	-	+	-	-	-	+	-	-	-
CRF2	2018	128	64	128	64	64	-	+	-	-	-	+	-	-	-
CRF4	2018	128	128	64	128	256	-	+	-	-	-	+	-	-	-
CRF5	2018	128	256	64	512	128	-	+	-	-	-	+	-	-	-
CRF7	2019	8	16	32	16	32	-	+	-	-	-	+	-	-	-
CRF8	2019	8	ND	ND	32	64	-	-	-	-	-	-	-	-	-
KLO7	2019	256	512	64	256	128	-	+	-	+	-	+	+	+	-
KLO5	2018	256	512	64	512	256	-	+	-	+	-	+	+	+	-
KLO3	2018	512	256	128	512	128	-	+	-	-	-	+	-	-	-
KLO2	2018	256	512	64	512	256	-	-	-	-	-	+	-	-	-
KLO1	2018	256	512	64	512	64	-	-	-	-	-	+	-	-	-
KLO6	2018	32	16	32	16	32	-	+	-	+	-	-	+	+	-
KLO4	2018	32	32	16	64	32	-	+	-	-	-	+	+	-	-
ERC1	2018	256	512	64	512	128	-	-	-	-	+	+	+	-	-
ERC3	2018	512	256	128	512	256	-	-	-	-	+	-	-	-	-
ERC2	2018	32	32	16	32	64	-	-	-	-	+	+	+	-	-
ERC4	2018	16	32	16	32	32	-	-	-	-	-	-	-	-	-
ERC5	2019	ND	16	ND	16	32	-	-	-	-	-	-	-	-	-
ERC6	2019	ND	8	ND	8	8	-	-	-	-	-	-	-	-	-
PSV2	2018	ND	32	64	32	64	+	-	-	-	-	+	-	-	-
PSV1	2018	16	32	ND	32	32	+	-	-	-	-	+	-	-	-

PSV3	2019	ND	ND	32	128	16	-	-	-	-	-	+	-	-	-
MAM1	2019	32	128	128	128	512	-	+	-	-	-	-	-	-	-
MAM3	2019	4	ND	32	16	64	-	-	-	-	-	-	-	-	-
MAM2	2019	ND	16	ND	64	16	-	-	-	-	-	-	-	-	-

Abbreviations used in Tables; EC- *Escherichia coli*, KLP- *Klebsiella pneumonia*, PLM- *Proteus mirabilis* ,CRF- *Citrobacter freundii*, KLO - *Klebsiella oxytoca*, ERC – *Enterobacter cloacae*, PSV- *Proteus vulgaris*, MAM- *Morganella morganii* , R- Resistance , S- Susceptible ,I –Intermediate Resistance , ND- Not done

Table S7. Distribution of aminoglycoside modifying genes in extended spectrum beta-lactamase producing *Enterobacterales* isolates and its comparison with minimum inhibitory concentrations of aminoglycosides tested.

Key for Isolates	Year of isolation	MICs of Aminoglycosides				AME encoding genes detected									
		GEN	TOB	AMK	K	*aac(3)-Ib*	*aac(3)-Ia*	*aac(3)-IIa*	*aac(6')-Ib*	*ant(2")-Ia*	*ant(3")-Ia*	*ant(4")-IIa*	*aph(3')-Ia*	*aph(3")-Ib*	*armA*
EC13	2018	1024	512	256	256	-	-	+	+	-	-	-	-	-	-
EC23	2019	1024	512	64	128	-	-	+	+	-	-	-	-	-	-
EC10	2018	1024	1024	64	128	-	-	+	+	-	+	+	+	+	-
EC18	2018	1024	1024	256	512	-	+	-	+	-	+	-	+	+	-
EC26	2019	1024	512	256	512	-	-	-	+	-	+	-	+	+	-
EC12	2018	512	128	64	128	-	-	+	+	-	-	-	-	-	-
EC21	2018	512	256	64	128	-	-	+	+	-	-	-	-	-	-
EC20	2018	512	256	64	256	-	-	+	+	-	-	-	-	-	-
EC9	2018	512	64	64	128	-	-	+	+	-	+	+	+	-	-
EC15	2018	512	256	64	128	+	-	+	+	-	+	+	+	+	-
EC17	2018	512	256	64	256	-	+	-	+	-	+	+	+	+	-
EC19	2018	512	256	128	512	-	+	-	+	-	+	-	+	+	-
EC24	2019	256	512	64	256	-	+	-	+	-	+	-	+	+	-
EC25	2019	256	512	64	512	-	+	-	+	-	+	-	+	+	-
EC16	2018	256	512	128	256	+	+	-	+	-	+	+	+	+	-
EC29	2019	128	256	64	512	-	-	-	+	-	+	-	-	+	-
EC22	2018	256	512	64	1024	-	-	+	+	-	-	-	-	-	-
EC11	2018	256	64	32	128	-	-	+	+	-	-	-	-	-	-
EC8	2018	256	128	128	64	-	-	+	+	-	+	+	+	-	-
EC14	2018	128	256	32	128	-	-	+	+	-	-	-	-	-	-
EC6	2018	128	64	32	32	-	-	+	+	-	-	-	-	-	-
EC4	2018	128	64	32	128	-	-	+	+	-	-	-	+	-	-
EC33	2019	128	64	32	32	-	-	+	+	-	-	-	+	-	-
EC3	2018	128	32	32	128	-	-	+	+	-	-	+	+	-	-
EC5	2018	128	32	32	32	-	-	+	+	-	-	+	+	-	-
EC27	2019	128	64	128	64	-	-	-	+	-	+	-	-	+	-

EC28	2019	128	128	64	128	-	-	-	+	-	+	-	-	+	-
EC7	2018	64	64	32	256	-	-	+	+	-	-	-	+	-	-
EC40	2019	32	32	64	64	-	-	-	-	-	+	-	-	+	-
EC32	2019	32	32	16	64	-	-	-	-	-	+	-	-	+	-
EC39	2019	32	16	32	64	-	-	-	-	-	+	-	-	+	-
EC36	2019	32	64	32	128	-	-	-	-	-	+	-	-	+	-
EC37	2019	32	32	ND	64	-	-	-	-	-	+	-	-	+	-
EC1	2018	32	32	ND	64	-	-	-	-	-	+	-	-	+	-
EC2	2018	16	32	ND	64	-	-	-	-	-	+	-	-	+	-
EC30	2019	32	64	ND	256	-	-	-	-	-	+	-	-	+	+
EC31	2019	32	16	ND	64	-	-	-	-	-	-	-	-	-	+
EC34	2019	32	16	ND	32	-	-	-	-	-	-	-	-	-	+
EC35	2019	32	16	ND	64	-	-	-	-	-	-	-	-	-	+
EC38	2019	32	16	ND	64	-	-	-	-	-	-	-	-	-	+
EC41	2019	ND	32	ND	128	-	-	-	-	-	-	-	-	-	-
EC42	2019	ND	16	ND	64	-	-	-	-	-	-	-	-	-	-
EC43	2019	ND	32	ND	128	-	-	-	-	-	-	-	-	-	-
EC44	2019	ND	64	ND	64	-	-	-	-	-	-	-	-	-	-
EC45	2019	ND	16	ND	64	-	-	-	-	-	-	-	-	-	-
KLP16	2018	1024	512	256	256	+	-	-	+	-	-	-	-	-	-
KLP12	2018	1024	512	64	1024	-	-	-	+	-	-	-	-	-	-
KLP27	2018	1024	1024	64	1024	-	-	+	+	+	+	-	+	+	-
KLP42	2018	1024	512	256	512	-	-	+	+	-	-	+	+	+	+
KLP25	2018	1024	1024	64	512	-	-	-	+	-	-	-	+	-	-
KLP38	2018	1024	512	256	1024	-	-	-	+	-	-	-		-	-
KLP17	2018	512	256	64	512	+	-	-	+	-	-	-		-	-
KLP4	2018	512	256	64	128	-	-	-	+	-	-	-	+	-	-
KLP23	2018	512	1024	256	512	-	-	+	+	+	+	-	+	-	-
KLP26	2018	512	64	64	32	-	-	+	+	+	+	-	+	+	-
KLP28	2018	512	256	64	512	-	-	+	+	+	+	-	+	+	-

KLP30	2018	512	256	64	512	-	-	+	+	-	+	-	+	+	-
KLP33	2018	512	256	128	128	-	-	+	+	-	+	-	+	+	+
KLP24	2018	512	64	64	128	-	-	-	+	-	-	-	+	-	-
KLP22	2018	128	512	128	1024	-	-	+	+	+	+	-	+	-	-
KLP8	2018	256	512	64	1024	+	-	-	+	-	-	-	-	-	-
KLP14	2018	512	256	64	128	-	-	-	+	-	-	-	-	-	-
KLP10	2018	512	256	64	512	-	-	-	+	-	-	-	-	-	-
KLP21	2018	512	256	128	128	-	-	-	+	-	-	-	-	-	-
KLP5	2018	256	128	128	256	-	-	-	+	-	-	-	+	-	-
KLP31	2018	256	512	64	1024	-	-	-	+	-	-	-	-	-	-
KLP35	2018	256	512	64	1024	-	-	-	+	-	-	-	-	-	-
KLP13	2018	256	512	128	512	-	-	-	+	-	-	-	-	-	-
KLP29	2018	256	512	128	256	-	-	+	+	-	+	-	+	+	-
KLP7	2018	256	64	16	32	-	-	-	+	-	+	-	+	-	-
KLP34	2018	256	512	64	512	-	-	+	+	-	+	-	+	+	+
KLP41	2018	256	512	64	1024	-	-	+	+	-	-	+	+	+	+
KLP3	2018	128	64	64	512	-	-	-	+	-	-	-	-	-	-
KLP32	2018	128	64	64	128	-	-	+	+	-	+	-	+	+	+
KLP43	2018	128	16	16	32	-	-	+	+	-	-	+	+	+	+
KLP18	2018	128	256	32	512	-	-	-	+	-	+	-	+	-	-
KLP2	2018	128	32	32	128	-	-	-	+	-	-	-	+	-	-
KLP9	2018	128	64	32	256	-	-	-	+	-	+	-	+	-	-
KLP15	2018	128	64	32	256	-	-	-	+	-	+	-	+	-	-
KLP19	2018	128	256	64	128	-	-	-	+	-	+	-	+	-	-
KLP20	2018	128	32	32	32	-	-	+	+	+	+	-	+	-	-
KLP37	2018	64	128	32	256	-	-	+	+	-	-	-	+	-	-
KLP11	2018	64	64	32	128	-	-	-	+	-	+	-	+	-	-
KLP1	2018	32	16	16	8	+	-	-	+	-	-	-	-	-	-
KLP44	2018	32	16	8	16	-	-	+	+	-	-	+	+	+	+
KLP40	2018	32	16	ND	8	-	-	-	+	-	-	-	-	-	-

KLP46	2018	32	16	ND	8	-	-	-	+	-	-	-	-	-	-
KLP45	2018	16	8	8	8	-	-	+	+	-	-	-	+	+	+
KLP36	2018	16	32	16	64	-	-	+	+	-	-	-	+	+	+
KLP6	2018	16	32	16	64	-	-	+	+	-	-	-	+	+	+
KLP39	2018	16	32	16	32	-	-	-	+	-	-	-	-	-	-
KLP47	2018	ND	32	ND	32	-	-	-	-	-	-	-	-	-	-
KLP48	2019	ND	16	ND	16	-	-	-	-	-	-	-	-	-	-
KLP49	2019	ND	16	ND	16	-	-	-	-	-	-	-	-	-	-
KLP50	2019	ND	8	ND	8	-	-	-	-	-	-	-	-	-	-
PLM1	2018	1024	256	128	ND	-	-	-	+	-	+	-	-	-	-
PLM3	2018	256	128	32	ND	-	-	-	+	-	-	-	-	-	-
PLM2	2018	16	32	16	64	-	-	+	+	-	+	+	+	+	-
PLM4	2018	16	16	ND	32	-	-	-	-	-	-	-	-	-	-
PLM5	2018	8	32	ND	16	-	-	-	-	-	-	-	-	-	-
PLM6	2018	16	ND	ND	ND	-	-	-	-	-	-	-	-	-	-
PLM7	2019	32	ND	ND	ND	-	-	-	-	-	-	-	-	-	-
CRF1	2018	512	256	64	256	+	+	-	+	-	-	-	-	-	-
CRF3	2018	512	256	64	512	+	-	-	-	-	-	-	-	-	-
CRF2	2018	128	512	128	256	+	-	-	+	-	-	-	-	-	-
CRF6	2018	32	128	16	64	-	-	+	-	-	+	-	-	-	-
CRF4	2018	128	64	64	128	-	-	+	-	-	+	-	-	-	-
CRF5	2018	16	64	32	128	-	-	+	-	-	+	-	-	-	-
CRF7	2019	ND	8	ND	ND	-	-	-	-	-	-	-	-	-	-
KLO1	2018	128	256	64	128	-	-	-	+	-	-	-	-	-	-
KLO2	2018	128	512	32	1024	-	-	+	+	-	-	-	-	+	-
KLO3	2018	64	128	32	256	-	-	-	+	-	-	-	-	-	-
KLO4	2018	16	32	16	16	+	-	+	+	+	+	-	+	+	-
KLO5	2018	ND	16	32	ND	-	-	-	-	-	-	-	-	-	-
KLO6	2018	ND	32	ND	32	-	-	-	-	-	-	-	-	-	-
ERC2	2018	256	512	64	1024	-	-	+	-	+	-	-	-	+	-

ERC1	2018	32	128	64	128	+	-	+	+	+	+	-	-	+	-
ERC3	2018	16	64	32	32	-	-	+	-	-	-	-	-	-	-
ERC4	2018	32	64	64	128	-	-	-	-	-	-	-	-	-	-
ERC5	2019	16	16	32	32	-	-	-	-	-	-	-	-	-	-
ERC6	2019	16	32	8	32	-	-	-	-	-	-	-	-	-	-
ERC7	2019	16	64	ND	32	-	-	-	-	-	-	-	-	-	-
ERC8	2019	8	ND	ND	ND	-	-	-	-	-	-	-	-	-	-
PSV1	2018	32	ND	ND	ND	-	-	-	-	-	+	-	-	-	-
PSV2	2018	ND	32	ND	16	-	-	-	-	-	-	-	-	-	-
PSV3	2019	ND	16	ND	32	-	-	-	-	-	-	-	-	-	-
MAM1	2019	32	128	16	256	-	-	+	+	-	-	-	-	-	-
MAM3	2019		32	32	64	-	-	-	-	-	-	-	-	-	-
MAM2	2019	16	ND	ND	ND	-	-	-	-	-	-	-	-	-	-

Abbreviations used in Tables ; EC- *Escherichia coli*, KLP- *Klebsiella pneumonia*, PLM- *Proteus mirabilis* ,CRF- *Citrobacter freundii*, KLO - *Klebsiella oxytoca*, ERC – *Enterobacter cloacae*, PSV- *Proteus vulgaris*, MAM- *Morganella morganii* , R- Resistance , S- Susceptible ,I –Intermediate Resistance , ND- Not done

Table S8. Distribution of trimethoprim-sulfamethoxazole (TMP-SMX) resistance genes in extended spectrum beta-lactamase producing *Enterobacterales* isolates and its comparison with minimum inhibitory concentrations of TMP-SMX tested.

Key for Isolates	Year of isolation		TMP-SMX Genes detected		
		MICs of TMP-SMX	*sul 1*	*sul 2*	*dfr A*
EC20	2018	16/304	+	-	+
EC14	2018	16/304	+	-	+
EC7	2018	16/304	+	-	+
EC4	2018	16/304	+	-	+
EC23	2019	16/304	+	-	-
EC33	2019	16/304	+	+	-
EC29	2019	16/304	+	+	-
EC15	2018	16/304	+	-	+
EC16	2018	16/304	+	-	+
EC19	2018	16/304	+	-	+
EC8	2018	16/304	+	-	+
EC9	2018	16/304	+	-	+
EC10	2018	16/304	+	-	+
EC25	2019	16/304	+	-	-
EC26	2019	16/304	+	-	-
EC30	2019	16/304	+	+	-
EC31	2019	16/304	+	+	-
EC35	2019	16/304	+	+	-
EC1	2018	16/304	+	-	+
EC2	2018	16/304	+	-	+
EC22	2018	16/304	+	-	-
EC38	2019	16/304	-	-	-
EC40	2019	16/304	-	-	-
EC43	2019	16/304	-	-	-
EC45	2019	16/304	-	-	-
EC52	2019	16/304	-	-	-

EC56	2019	16/304	-	-	-
EC13	2018	8/152	+	-	+
EC21	2018	8/152	+	-	-
EC11	2018	8/152	+	-	+
EC28	2019	8/152	+	+	-
EC17	2018	8/152	+	-	+
EC5	2018	8/152	+	-	+
EC3	2018	8/152	+	-	+
EC34	2019	8/152	+	+	-
EC12	2018	4l76	+	-	+
EC6	2018	4l76	+	-	+
EC27	2019	4l76	+	+	-
EC18	2018	4l76	+	-	+
EC24	2019	4l76	+	-	-
EC32	2019	4l76	+	+	-
EC55	2019	4l76	-	-	-
EC42	2019	4l76	-	-	-
EC36	2019	4l76	-	-	-
EC37	2019	8/152	-	-	-
EC41	2019	8/152	-	-	-
EC44	2019	8/152	-	-	-
EC48	2019	8/152	-	-	-
EC51	2019	8/152	-	-	-
EC53	2019	8/152	-	-	-
EC54	2019	8/152	-	-	-
EC57	2019	8/152	-	-	-
EC49	2019	4l76	-	-	-
EC50	2019	4l76	-	-	-
EC46	2019	4l76	-	-	-
EC39	2019	4l76	-	-	-

KLP8	2018	16/304	+	-	+
KLP12	2018	16/304	+	-	+
KLP1	2018	16/304	+	+	+
KLP7	2018	16/304	+	-	+
KLP11	2018	16/304	+	-	+
KLP15	2018	16/304	+	-	+
KLP18	2018	16/304	+	-	+
KLP22	2018	16/304	+	-	+
KLP23	2018	16/304	+	-	+
KLP26	2018	16/304	+	-	+
KLP29	2018	16/304	+	-	-
KLP30	2018	16/304	+	-	-
KLP34	2018	16/304	+	-	-
KLP36	2018	16/304	+	-	-
KLP43	2018	16/304	+	-	-
KLP44	2018	16/304	+	-	-
KLP41	2018	16/304	+	-	-
KLP4	2018	16/304	+	+	+
KLP9	2018	16/304	+	-	+
KLP6	2018	16/304	+	+	+
KLP37	2018	16/304	+	-	-
KLP38	2018	16/304	+	-	-
KLP39	2018	16/304	+	-	-
KLP10	2018	16/304	+	-	+
KLP21	2018	16/304	+	-	+
KLP31	2018	16/304	+	-	-
KLP35	2018	16/304	+	-	-
KLP3	2018	16/304	+	+	+
KLP46	2018	16/304	+	-	-
KLP48	2019	16/304	-	-	-

KLP50	2019	16/304	-	-	-
KLP52	2019	16/304	-	-	-
KLP53	2019	16/304	-	-	-
KLP55	2019	16/304	-	-	-
KLP56	2019	16/304	-	-	-
KLP57	2019	16/304	-	-	-
KLP58	2019	16/304	-	-	-
KLP60	2019	16/304	-	-	-
KLP61	2019	16/304	-	-	-
KLP62	2019	16/304	-	-	-
KLP64	2019	16/304	-	-	-
KLP66	2019	16/304	-	-	-
KLP67	2019	16/304	-	-	-
KLP16	2018	8/152	+	-	+
KLP20	2018	8/152	+	-	+
KLP27	2018	8/152	+	-	+
KLP32	2018	8/152	+	-	-
KLP42	2018	8/152	+	-	-
KLP5	2018	8/152	+	+	+
KLP14	2018	8/152	+	-	+
KLP40	2018	8/152	+	-	-
KLP24	2018	8/152	+	-	+
KLP49	2019	8/152	-	-	-
KLP51	2019	8/152	-	-	-
KLP54	2019	8/152	-	-	-
KLP65	2019	8/152	-	-	-
KLP17	2018	4l76	+	-	+
KLP19	2018	4l76	+	-	+
KLP28	2018	4l76	+	-	+
KLP33	2018	4l76	+	-	-

KLP45	2018	4l76	+	-	-
KLP13	2018	4l76	+	-	+
KLP2	2018	4l76	+	+	+
KLP25	2018	4l76	+	-	+
KLP47	2018	4l76	-	-	-
KLP59	2019	4l76	-	-	-
KLP63	2019	4l76	-	-	-
PLM6	2018	16/304	+	-	-
PLM1	2018	16/304	+	-	+
PLM3	2018	16/304	+	-	-
PLM7	2019	16/304	-	-	-
PLM10	2019	16/304	-	-	-
PLM4	2018	8/152	+	-	-
PLM5	2018	8/152	+	-	-
PLM9	2019	8/152	-	-	-
PLM12	2019	8/152	-	-	-
PLM2	2018	4l76	+	-	+
PLM8	2019	4l76	-	-	-
PLM11	2019	4l76	-	-	-
CRF1	2018	16/304	+	-	+
CRF5	2018	16/304	+	-	-
CRF7	2019	16/304	+	-	-
CRF9	2019	16/304	-	-	-
CRF11	2019	16/304	-	-	-
CRF3	2018	8/152	+	-	+
CRF6	2018	8/152	+	-	-
CRF4	2018	8/152	+	-	+
CRF10	2019	8/152	-	-	-
CRF2	2018	4l76	+	-	+
CRF8	2019	4l76	-	-	-

KLO6	2018	16/304	+	-	-
KLO4	2018	16/304	+	-	-
KLO5	2018	16/304	+	-	-
KLO1	2018	16/304	+	+	+
KLO3	2018	8/152	+	-	-
KLO7	2019	8/152	-	-	-
KLO8	2019	8/152	-	-	-
KLO2	2018	4l76	+	-	-
KLO9	2019	4l76	-	-	-
ERC5	2019	16/304	+	-	-
ERC2	2018	16/304	+	-	+
ERC6	2019	16/304	-	-	-
ERC7	2019	16/304	-	-	-
ERC1	2018	8/152	+	+	+
ERC3	2018	8/152	+	-	-
ERC4	2018	8/152	+	-	-
ERC8	2019	8/152	-	-	-
ERC9	2019	8/152	-	-	-
PSV1	2018	8/152	+	-	+
PSV2	2018	4l76	+	-	-
MAM1	2019	16/304	+	-	+
MAM2	2019	4l76	-	-	-
MAM3	2019	4l76	-	-	-

Abbreviations used in Tables ; EC- *Escherichia coli*, KLP- *Klebsiella pneumonia,* PLM- *Proteus mirabilis* ,CRF- *Citrobacter freundii*, KLO - *Klebsiella oxytoca,* ERC – *Enterobacter cloacae*, PSV- *Proteus vulgaris*, MAM- *Morganella morganii* , R- Resistance , S- Susceptible ,I –Intermediate Resistance , ND- Not done
